# Hydroxybenzothiazoles as New Nonsteroidal Inhibitors of 17β-Hydroxysteroid Dehydrogenase Type 1 (17β-HSD1)

**DOI:** 10.1371/journal.pone.0029252

**Published:** 2012-01-05

**Authors:** Alessandro Spadaro, Matthias Negri, Sandrine Marchais-Oberwinkler, Emmanuel Bey, Martin Frotscher

**Affiliations:** 1 Pharmaceutical and Medicinal Chemistry, Saarland University, Saarbrücken, Germany; 2 ElexoPharm GmbH, Saarbrücken, Germany; 3 Helmholtz Institute for Pharmaceutical Research Saarland (HIPS), Saarbrücken, Germany; National Cancer Center, Japan

## Abstract

17β-estradiol (E2), the most potent estrogen in humans, known to be involved in the development and progession of estrogen-dependent diseases (EDD) like breast cancer and endometriosis. 17β-HSD1, which catalyses the reduction of the weak estrogen estrone (E1) to E2, is often overexpressed in breast cancer and endometriotic tissues. An inhibition of 17β-HSD1 could selectively reduce the local E2-level thus allowing for a novel, targeted approach in the treatment of EDD. Continuing our search for new nonsteroidal 17β-HSD1 inhibitors, a novel pharmacophore model was derived from crystallographic data and used for the virtual screening of a small library of compounds. Subsequent experimental verification of the virtual hits led to the identification of the moderately active compound **5**. Rigidification and further structure modifications resulted in the discovery of a novel class of 17β-HSD1 inhibitors bearing a benzothiazole-scaffold linked to a phenyl ring via keto- or amide-bridge. Their putative binding modes were investigated by correlating their biological data with features of the pharmacophore model. The most active keto-derivative **6** shows IC_50_-values in the nanomolar range for the transformation of E1 to E2 by 17β-HSD1, reasonable selectivity against 17β-HSD2 but pronounced affinity to the estrogen receptors (ERs). On the other hand, the best amide-derivative **21** shows only medium 17β-HSD1 inhibitory activity at the target enzyme as well as fair selectivity against 17β-HSD2 and ERs. The compounds **6** and **21** can be regarded as first benzothiazole-type 17β-HSD1 inhibitors for the development of potential therapeutics.

## Introduction

Estrogens are important steroidal hormones which exert different physiological functions. The main beneficial effects include their role in programming the breast and uterus for sexual reproduction [1], controlling cholesterol production in ways that limit the build-up of plaque in the coronary arteries [2], and preserving bone strength by helping to maintain the proper balance between bone build-up and breakdown [3]–[4]. Among female sex hormones, 17β-estradiol (E2) is the most potent estrogen carrying out its action either via transactivation of estrogen receptors (ERs) [5] or by stimulating nongenomic effects via the MAPK (mitogen-activated protein kinase) signaling pathway [6]. In addition to its important beneficial effects, however, E2 can also cause serious problems arising from its ability to promote the cell proliferation in breast and uterus. Although this is one of the normal functions of estrogen in the body, it can also increase the risk of estrogen dependent diseases (EDD), like breast cancer, endometriosis and endometrial hyperplasia [7]–[10]. Suppression of estrogenic effects is consequently a major therapeutic approach. This is proved by routine clinic use of different endocrine therapies, for instance with GnRH analogues, SERMs (selective estrogen receptor modulators), antiestrogens, and aromatase inhibitors [11]–[13] for the prevention as well as the adjuvant treatment of breast cancer. However, all these therapeutics systemically lower estrogen hormone action and may cause significant side effects such as osteoporosis, thrombosis, stroke and endometrial cancer [14]–[16]. Thus, a new approach, which aims at affecting predominantly the intracellular E2 production in the diseased tissues (intracrine approach), would consequently be a very beneficial improvement for the treatment of EDD. Such a therapeutic strategy has already been shown to be effective in androgen dependent diseases like benign prostate hyperplasia by using 5α-reductase inhibitors [17]–[21].

17β-HSD1, which is responsible for the intracellular NAD(P)H-dependent conversion of the weak estrone E1 into the highly potent estrogen E2, was found overexpressed at mRNA level in breast cancer cells [22]–[24] and endometriosis [25]. Inhibition of this enzyme is therefore regarded as a novel intracrine strategy in EDD treatment with the prospect of avoiding the systemic side effects of the existing endocrine therapies. Although to date no candidate has entered clinical trials, the ability of 17β-HSD1 inhibitors to reduce the E1 induced tumor growth has been shown using different animal models, indicating that the 17β-HSD1 enzyme is a suitable target for the treatment of breast cancer [26]–[28]. The same effect was also demonstrated by Day et al. [28], Laplante et al. [29] and Kruchten et al. [30] using *in vitro* proliferation assays.

In order not to counteract the therapeutic efficacy of 17β-HSD1 inhibitors it is important that the compounds are selective against 17β-hydroxysteroid dehydrogenase type 2 (17β-HSD2). This enzyme catalyses the reverse reaction (oxidation of E2 to E1), thus playing a protective role against enhanced E2 formation in the diseased estrogen dependent tissues. Potent and selective 17β-HSD2 inhibitors for the treatment of osteoporosis were recently reported [31]–[32]. Additionally, to avoid intrinsic estrogenic and systemic effects, the inhibitors should not show affinity to the estrogen receptors α and β.

**Figure 1:**
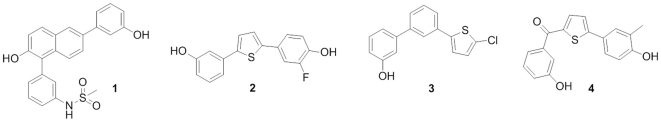
Nonsteroidal 17β-HSD1 inhibitors published by our group.

Several classes of 17β-HSD1 inhibitors have been described in the last years [33]–[47], most of them having a steroidal structure. During the past decade, our group reported on four different classes of nonsteroidal 17β-HSD1 inhibitors [48]–[58]. Compounds **1–4** (Figure 1) exhibit IC_50_ values toward 17β-HSD1 in the nanomolar range and high selectivity against 17β-HSD2 and the ERs in our biological screening system [59].

In our search for new nonsteroidal 17β-HSD1 inhibitors that are structurally different from those previously described, an *in silico* screening of an in-house compound library was performed using a pharmacophore model derived from crystallographic data. Upon experimental validation, a virtual hit could be identified as a moderately active inhibitor of 17β-HSD1 (Table S1, compound **5**); structural optimization led to the discovery of benzothiazoles as novel, potent inhibitors of the target enzyme with good biological activity *in vitro*. Further computational studies were performed to better understand the favourable interactions achieved by these inhibitors in the active site.

## Materials and Methods

### Pharmacophore model

Although up to now more than twenty crystallographic structures of human 17β-HSD1 are available in the Protein Data Bank (PDB) as apoform, binary or ternary complex (with steroidal ligand and/or cosubstrate), X-ray based information about protein-ligand interactions of the enzyme with nonsteroidal inhibitors is not available. Furthermore, the steroidal binding pocket in these crystal structures displays differences in terms of size and geometry due to a pronounced flexibility of some parts of its surroundings [60]. Therefore, a simple virtual screening strategy such as random selection of one crystal structure to perform docking studies was considered unsuitable to the search for new hits. As *in silico* screening tool, a new pharmacophore model for 17β-HSD1, based on cocrystallized ligands and with some additional protein structure information, was built and a ligand-based approach was followed instead.

**Figure 2:**
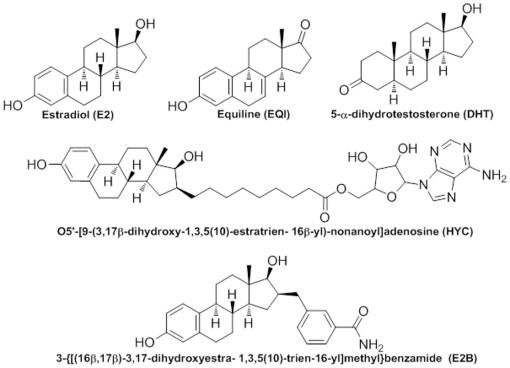
Steroidal ligands co-crystallized with 17β-HSD1. The five steroidal ligands cocrystalized with 17β-HSD1s that were used to build the pharmacophore model. Structural information was taken from the protein data bank(PDB-ID: 1a27, 1equ, 1dht, 1i5r, and 3hb5, respectively).

Five diverse 17β-HSD1 crystal structures (PDB-ID: 1a27, 1equ, 1dht, 1i5r, 3hb5) [61]–[65] were superimposed (backbone root mean square deviation (RMSD) of 0.7 Å) and the cocrystallized steroidal ligands E2, EQI, DHT, HYC and E2B, respectively, (Figure 2) were used to build the pharmacophore model using the Molecular Operating Environment (MOE; www.chemcomp.com) software.

The five crystal structures were chosen to cover most of the chemical space occupied by their 17β-HSD1 ligands, and both the presence of the cosubstrate (NADP^+^/NADPH) and a good resolution were considered important for their selection as the pharmacophore model should integrate both ligand- and protein-derived information, gained from the analysis of different crystal structures.

Superimposition of the mentioned 3D complexes (ligands and proteins) enabled us to define the pharmacophore features of both the ligands and of the constant regions of the protein, involved in ligand-protein interaction. While the selection of the ligand-derived features was focused on the slightly different chemical properties of substrates and inhibitors, the protein-derived features were chosen considering “rigid” active site residues (all atom RMSD for all crystal structures<0.5 Å) as well as amino acids important for the enzymatic activity. Furthermore, the flexible βFαG'-loop (residues 187–196) [60] lining the active site was excluded, whereas additional donor/acceptor features of the cofactor (NADP^+^) were considered.

**Figure 3:**
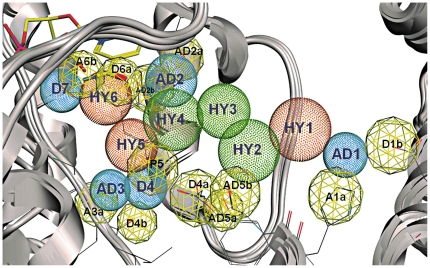
Pharmacophore model. The pharmacophoric features derived from the ligands are rendered as dotted spheres and are color-coded: dark orange for aromatic ring and aromatic ring projection (**HY1** and **HY5**), green for hydrophobic regions (**HY2-HY4**) and magenta for acceptor and donor atom features (**AD1-AD3** and **D4**). The identified aromatic ring projection **HY6** as well as the donor projection feature **D7** is not exploited by steroidal inhibitors. The protein-derived acceptor or donor features (**A1a**, **D1b**, **AD2a**, **AD2b**, **A3a**, **D4a**, **D4b**, **AD5a**, **AD5b**, **D6a** and **A6b**) and the aromatic ring projection **P5** are depicted as yellow, meshed spheres.

Thus, on the ligand side the pharmacophore model (Figure 3) consists of five hydrophobic/aromatic features **HY1-HY5** (four from steroid scaffold and one from E2B/HYC, respectively), one aromatic ring projection **P5** (associated to **HY5;** used to direct the ligand placement in the pharmacophore screen), three H-bond acceptor-and-donor **AD1-AD3** (two from the steroid scaffold and one from E2B) and one H-bond donor **D4** (corresponding to the NH_2_ of the amide in E2B).

Nine acceptor (**A**) or donor (**D**) feature projections were derived from the protein and were used to direct the ligand orientation in the pharmacophore screening (projections indicate putative protein binding partners; the number indicate the the ligand feature, while the small letters a and b describe the inverse H-bonding properties of residues involved in a common network, e.g. **A1a** is a donor and **D1b** is an acceptor, and both interact with **AD1**): **A1a** - His221, **D1b** - Glu282, **AD2a** - Ser142, **AD2b** - Tyr155, **A3a** - Leu96, **D4a** - Asn152, **D4b** - Leu95, **AD5a** - Ser222, **AD5b** - Tyr218. In addition, four features were also retained from the cofactor NADP(H): **A6b** and **D6a** from the amide moiety, **HY6** as aromatic ring projection from the nicotinamide ring (potential interaction site of the ligand with Tyr155 and cofactor), and **D7** as acceptor projection (phosphate group of the cofactor). More geometric properties of the pharmacophore are listed in Table S2.

This pharmacophore model, comprising 23 features, was now used to screen a small *in house* library (approximately forty thiazole derivatives with molecular weight in the range of 150–350; structures are given in Table S3) and the virtual hits were experimentally validated. A partial match strategy was adopted for the screening, in which the molecules are left free to be placed into the pharmacophore and only virtual hits are considered that cover at least six features.

### Chemistry

For the sake of clarity, IUPAC nomenclature is not strictly followed except for the experimental section (see File S1) where the correct IUPAC names are given.

**Figure 4:**
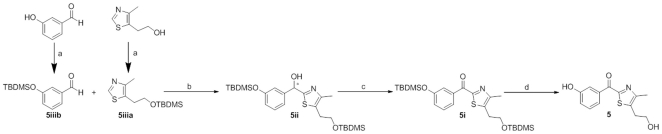
Synthesis of compound 5. Reagents and conditions: (a) TBDMSiCl, imidazole, DMF, rt, 20 h; (b) 1) nBuLi, anhydrous THF, −70°C, 1 h; 2) **5iib**, anhydrous THF, −15°C, 90 min; (c) SIBX, anhydrous THF, 0°C to 60°C, 20 h; (d) TBAF, THF, rt, 2 h.

The synthesis of the thiazolyl derivative **5** was performed as shown in Figure 4 starting from the commercially available 2-(4-methyl-1,3-thiazol-5-yl)ethanol and 3-hydroxybenzaldehyde.

To avoid side reactions due to the presence of the free hydroxy groups, they were reacted with *tert*-butyldimethylsilyl chloride and imidazole in DMF at room temperature overnight [66] to afford **5iiia** and **5iiib**, respectively. Then nucleophilic addition [67] of **5iiia** (after *in situ* lithiation in the 2-position) to **5iiib** in THF at −15°C for 90 minutes yielded the secondary alcohol **5ii**. The latter was oxidized to the carbonyl derivative **5i** using stabilized 2-iodoxybenzoic acid (SIBX) as oxidative reagent in THF at 60°C [68] prior to the removal of both silyl protecting groups under mild basic conditions (TBAF in THF at room temperature for 2 hours) [69] to afford compound **5**.

**Figure 5:**
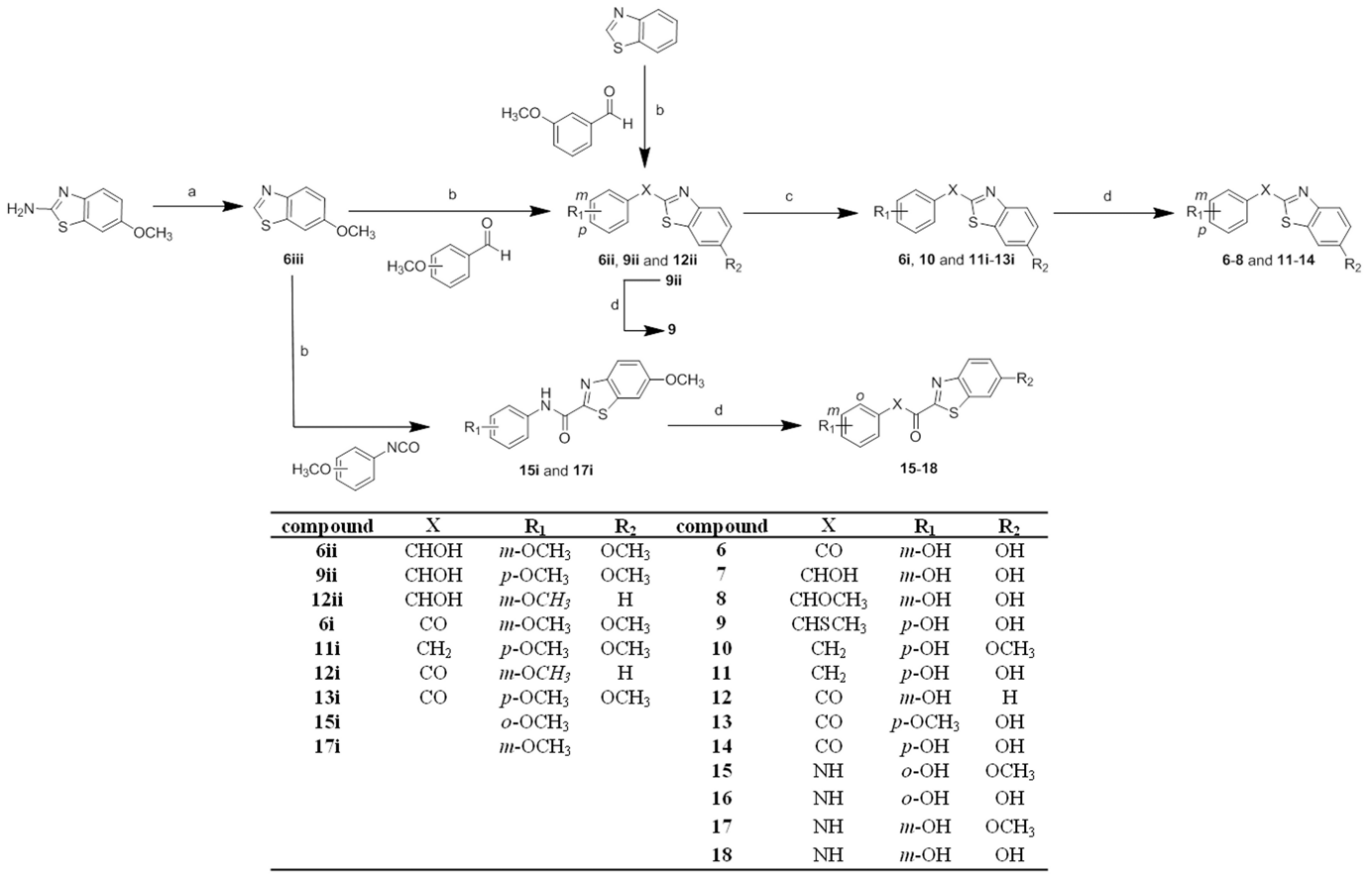
Synthesis of compounds 6–18. Reagents and conditions: (a) 1) NaNO_2_, H_3_PO_4_ (85%), −10°C, 20 min, 2) H_3_PO_2_, H_3_PO_4_ (85%), −10°C to rt, 20 h; (b) 1) nBuLi, anhydrous THF, −70°C to −20°C, 1 h, 2) for **6ii** and **12ii**: *m*-methoxybenzaldehyde, for **9ii**: *p*-methoxybenzaldehyde, for **15i**: *o*-methoxybenzoisocyanate, for **17i**: *m*-methoxybenzoisocyanate, anhydrous THF, −15°C, 90 min; (c) for **6i**, **12i** and **13i**, SIBX, anhydrous THF, 0°C to 60°C, 20 h; for **10** and **11i**: TMSiCl, NaI, CH_3_CN, reflux, 20 h; (d) for **6–9** and **15–18**: BF_3_S(CH_3_)_2_, anhydrous CH_2_Cl_2_, rt, 20 h; for compounds **11–14**, pyridinium hydrochloride, 220°C, 4 h.

The benzothiazolyl derivatives **6–11** and **13–18** were synthesized as shown in Figure 5.

The commercially available 6-methoxy-benzothiazol-2-yl amine was reduced to **6iii** in the first step via a previously described diazotation and subsequent reductive elimination of nitrogen [70] as follows: 6-methoxy-benzothiazol-2-yl amine was first dissolved in 85% phosphoric acid under gentle heating and then cooled to −10°C. Then, an aqueous solution of NaNO_2_ was slowly added to yield the diazonium salt. The latter was subsequently transformed *in situ* to **6iii** by adding the reaction mixture to chilled 50% aqueous phosphonic acid (0°C) and allowing the temperature to rise to room temperature overnight. The thus obtained intermediate **6iii** was lithiated in the 2-position and subjected to nucleophilic addition to 3-methoxybenzaldehyde or 4-methoxybenzaldehyde, respectively, in THF at −15°C for 90 minutes, to afford the secondary alcohols **6ii** and **9ii**. The same method was used for the synthesis of **12ii**, starting from the commercially available benzothiazole and 3-methoxybenzaldehyde, as well as for the preparation of the amides **15i** and **17i**, starting from **6iii** and 2-methoxybenzoisocyanate or 3-methoxybenzoisocyanate, respectively. The secondary alcohols **6ii**, **9ii** and **12ii** were oxidized to the corresponding carbonyl derivatives **6i**, **12i** and **13i**. The cleavage of the methoxy groups of **9ii** with BF_3_S(CH_3_)_2_ in anhydrous CH_2_Cl_2_ at room temperature for 20 hours [49] took place with concomitant formation of the thioether derivative **9**. The reduction of **9ii** using trimethyl silyl chloride and NaI in acetonitrile [71] led to the formation of the desired compound **11i** and an additional product (**10**), lacking one of the two methoxy groups . The reaction of **6i** with BF_3_S(CH_3_)_2_ led to the formation of the desired compound **6** and two additional reduced products (**7** and **8**). The ether cleavage of the methoxy groups of compounds **11i–13i** was carried out by using pyridinium hydrochloride at 220°C for 4 hours [49] to afford compounds **11–14**. This latter method proved to be very efficient for ketones. The cleavage of the methoxy groups of **15i** and **17i** with BF_3_S(CH_3_)_2_ gave access to the amide derivatives **15–18**. For some substrates the ether cleavage could not be driven to completeness. In those cases the formation of both monomethoxy derivatives was observed but only one could be isolated in the purification step.

**Figure 6:**
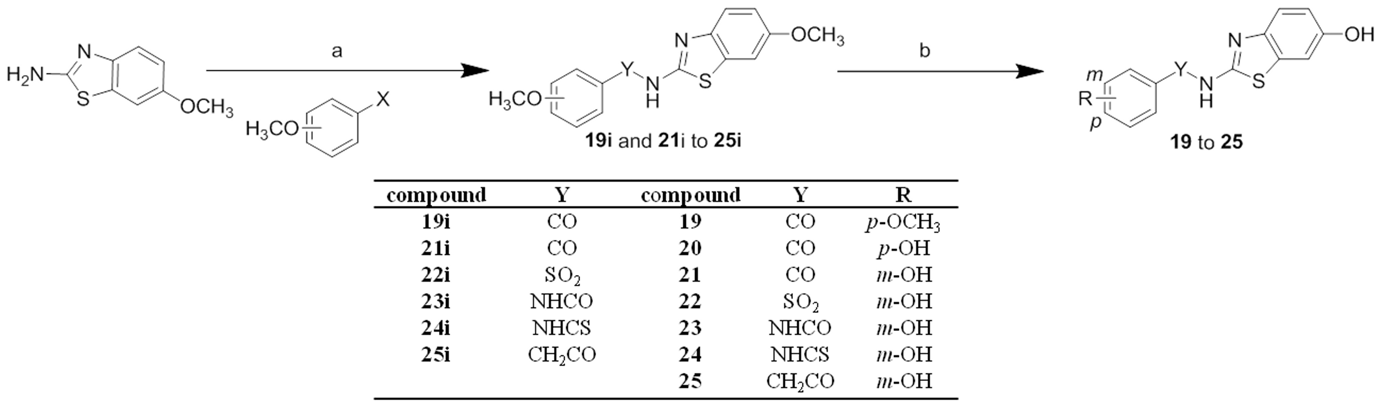
Synthesis of compounds 19–25. Reagents and conditions: (a) pyridine, 100°C, 20 h, for **19i**: *p*-methoxybenzoylchloride, for **21i**: *m*-methoxybenzoylchloride, for **22i**: *m*-methoxyphenylsulfochloride, for compound **23i**: *m*-methoxybenzoisocyanate, for **24i**: *m*-methoxybenzoisothiocyanate, for compound **25i**: *m*-methoxybenzylchloride; (b) for **19–21**, BF_3_S(CH_3_)_2_, anhydrous CH_2_Cl_2_, rt, 20 h; for **22–25**, BBr_3_, CH_2_Cl_2_, −78°C to rt, 20 h.

The benzamides (**19–21**), benzenesulfonamide (**22**), urea (**23**), thiourea (**24**) and acetamide (**25**) derivatives were afforded in a common two-steps synthetic pattern (Figure 6).

Compounds **19i** and **21i–25i** were synthesised via amide coupling [72] starting from the commercially available 6-methoxybenzothiazol-2-ylamine and 4-methoxybenzoyl chloride, 3-methoxybenzoyl chloride, 3-methoxysulfonyl chloride, 3-methoxybenzoisocyanate, 3-methoxybenzoisothiocyanate and 3-methoxyphenyl acetyl chloride, respectively. The ether cleavage of the methoxy groups was carried out to yield compounds **19–25**. Similarly to the ether cleavage of the amides, BF_3_S(CH_3_)_2_ was successfully applied in the case of retro amides (**19–21**). BBr_3_ was instead used for the synthesis of compounds **22–25**.

Fully described reactions conditions, NMR spectroscopic data and purity data for the final compounds using LC/MS are available in File S1.

### Biological assays

#### 1) 17β-HSD1 cell free assay

In the cell free assay, the placental cytosolic enzyme was used. Tritiated E1 (final concentration: 500 nM) was incubated with 17β-HSD1, NADH (500 µM), and inhibitor as previously described [59]. After high performance liquid chromatography (HPLC) separation of substrate and product, the amount of labeled E2 formed was quantified. The hybrid inhibitor (**EM-1745**) evaluated using recombinant protein and NADPH as cosubstrate in a cell free assay as described by Poirier et al. [64] was used as external reference and gave similar values as described (IC_50_ = 52 nM). Compounds showing less than 10% inhibition at 1 µM were considered to be inactive. IC_50_ values were determined for compounds showing more than 70% inhibition at 1 µM. Compound **2** (Figure 1), identified in a previous study [51], was used as internal reference (IC_50_ = 8 nM).

#### 2) 17β-HSD2 cell free assay

Inhibition of 17β-HSD2 was determined for compounds showing 17β-HSD1 inhibitory activity of 70% at 1 µM using an established assay [59] similar to the 17β-HSD1 test and a selectivity factor (SF = IC_50_(17β-HSD2)/IC_50_(17β-HSD1)) was determined. Placental microsomal 17β-HSD2 was incubated with tritiated E2 (final concentration: 500 nM) in the presence of NAD^+^ (1500 µM) and inhibitor. Separation and quantification of labeled substrate (E2) and product (E1) was performed by HPLC using radiodetection.

#### 3) ER binding affinity assay

The binding affinities to the ERs of the most interesting compounds of this study were determined using recombinant human protein (0.25 pmol of ERα or ERβ, respectively) in a previously described competition assay [59] applying [^3^H]E2 (10 nM) and hydroxyapatite.

#### 4) Intracellular potency

The intracellular potency of the most selective compound to inhibit E2 formation was evaluated as previously described [59] using the T47-D [30] cell line (obtained from ECACC, Salisbury) that expresses 17β-HSD1 and - to a much lesser extent -17β-HSD2 [73].

Fully described procedures regarding the biological assays are available as File S1.

## Results

### 1) Hit identification and optimization

**Figure 7:**
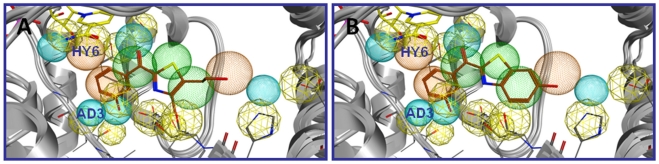
Compounds 5 (A) and 6 (B) mapped to the pharmacophore model.

[5-(2-hydroxyethyl)-4-methyl-1,3-thiazol-2-yl](3-hydroxyphenyl)methanone) (**5**) resulted to be the most potent hit, exploiting the following six features: **HY2-HY5**, **AD2** and **D4** (Figure 7A).

**Figure 8:**
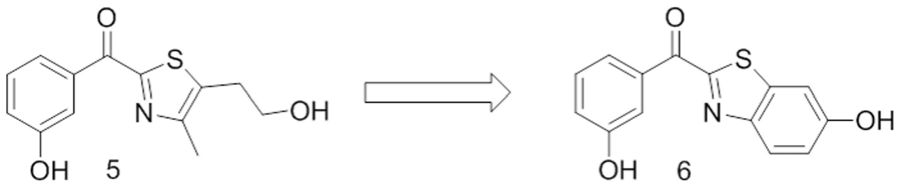
Rigidification strategy.

Applying the strategy of rigidification (decrease conformational degrees of freedom), compound **6** was designed which is characterized by a ring closure of the flexible hydroxyethyl chain of compound **5**, leading to a benzothiazole moiety linked via a carbonyl bridge in the 2 position to 3-hydroxy-phenyl ring (see Figure 8).

Interestingly, in a subsequent docking experiment, also compound **6**, the rigidified analogue of **5**, was found to match six pharmacophoric features (Figure 7B). Its superimposition with equilin, one of the steroidal ligands used to build the pharmacophore model, shows that the phenyl ring of the benzothiazole moiety can mimic the steroidal B ring (see Figure S1).

The carbonyl bridge between the two aromatic rings in compound **6** bearing a sp^2^ trigonal geometry allows for electronic delocalization (conjugation), and the oxygen atom may accept two H-bonds. To investigate the influence of these features on the 17β-HSD1 inhibitory activity, compounds **7–11** (Table S1), bearing a sp^3^ tetrahedral bridge, were synthesized.

The carbonyl bridge was also replaced by more spacious functional groups, such as amide (i.e. **18**), retro amide (i.e. **21**), sulfonamide (**22**), urea (**23**), thiourea (**24**) and benzyl amide (**25**) to inquire whether such a modification could allow the compounds to interact with different or additional regions of the binding cleft.

### 2) Activity: Inhibition of human 17β-HSD1

The inhibition values of the test compounds are shown in Table S1. The rigidified benzothiazole derivative **6** was far more potent towards the target enzyme (91% at 1 µM, IC_50_ = 44 nM) than **5** (34% at 1 µM) and thus turned out to be a very promising scaffold taking into account also its low molecular weight (271 g/mol).

The inhibitory potency of the compounds is strongly dependent on length and type of the bridge connecting the aromatic moieties. The replacement of the flat sp^2^ bridge (methanone in compound **6**) by a tetrahedral one, as present in the alcohol **7**, its methyl ether **8**, the corresponding thioether **9**, and the methylene compound **11**, turned out to be deleterious for the inhibitory activity at the target enzyme. Compounds **7**–**9** showed activities of 28%, 13% and 7%, respectively, at 1 µM concentration. The loss of potency observed for these compounds as well as for compounds **10** and **11** seems to be induced by the bridge geometry and not by the H-bonding properties, since a hydroxy- (acceptor and donor), a methoxymethyl- (acceptor only), and a methylene-group (neither acceptor nor donor) all led to a decrease in activity, compared to the carbonyl group.

As the keto bridge appeared to be the most appropriate functionality, it was maintained as starting point for further structural modifications. Compound **12**, without hydroxy group in the 6-position of the benzothiazole moiety, was designed to evaluate the importance of this functional group (**6i**) and showed 8 fold lower inhibitory activity than **6** (see Table S1). Furthermore, to investigate whether either a methoxy or a hydroxy group in *para*-positionon of the phenyl moiety of compound **6** could exploit the pharmacophoric feature **AD3** (see Figure 7B) compounds **13** and **14** were synthesized. The lower inhibitory activity of the *para*-methoxy derivative **13** (27% at 1 µM) compared to that of the the hydroxy compound **14** (85% at 1 µM, IC_50_ = 243 nM) points to the importance of an H-bonding donor in this position; there is, however, a decrease of activity when the hydroxy group is shifted from the *meta*- (compound **6**, IC_50_ = 44 nM) to the *para*-position (compound **14**).

Compounds with two bridging atoms between the hydroxyphenyl- and the hydroxybenzothiazole moieties, like amide **18**, retroamide **21**, and sulfonamide **22**, were also synthesized. In the amide series, both the H-bonding properties as well as the sp^2^ geometry turned out to be discriminating factors for 17β-HSD1 inhibitory activity. The introduction of an amide bridge in the place of the keto function results in a significantly decreased inhibitory potency (compound **18**: 40% inhibition at 1 µM *vs* compound **6**: IC_50_ = 44 nM). This is even more pronounced in the case of the sulfonamide derivative **22** which is inactive. Replacement with retro amide (leading to compound **21**), however, gives only a slight decrease of inhibitory activity (**21**: IC_50_ = 243 nM *vs* **6**: IC_50_ = 44 nM).

Changing the hydroxy substitution pattern (**16** and **20**) as well as replacement of –OH with methoxy (**15**, **17** and **19**) are modifications detrimental for activity, independent on the nature of the bridge (amide or retroamide).

The extension of the bridge to three units resulted in the inactive urea **23** and benzylamide **25**. Interestingly the thiourea **24** showed a moderate inhibitory activity.

### 3) Selectivity: Inhibition of 17β-HSD2 and affinities to the estrogen receptors α and β

In order to gain insight into the selectivity of the most active compounds, inhibition of 17β-HSD2 and the relative binding affinities to the estrogen receptors α and β were determined. Since 17β-HSD2 catalyzes the inactivation of E2 into E1, inhibitory activity toward this enzyme must be avoided. IC_50_ values and selectivity factors are presented in Table S4.

Among the series bearing the keto bridge, compound **6** showed the best selectivity factor (24 fold more active on 17β-HSD1 than 17β-HSD2). The absence of hydroxy on the benzothiazole (**12**) or moving the *meta* hydroxyl group of **6** to the *para*-position (compound **14)** leads to a drop in selectivity. Furthermore, compound **21**, bearing the retro amide bridge, shows a higher selectivity factor (SF = 38) than compound **18** (amide bridge, SF = 3) as well as compound **6** (carbonyl bridge, SF = 24).

The ER binding affinities of the most interesting compounds of this study (**6** and **21**) are shown in Table 1.

**Table 1:** Binding affinities for the human estrogen receptors α and β.

	RBA^a^ (%)	
Compound	ERα^b^	ERβ^b^
**6**	1<RBA<10	0.1<RBA<1
**21**	0.01<RBA<0.1	RBA<0.001

a RBA (relative binding affinity). E2: 100%, mean values of three determinations, standard deviations less than 10%.

b Human recombinant protein, incubation with 10 nM [^3^H]E2 and inhibitor for 1 h.

Compound **6** displays considerable affinities to both estrogen receptors, whereas compound **21** shows marginal affinities (RBA lower than 0.1% for ERα and lower than 0.001% for ERβ).

### 4) Further biological evaluation

The ability of **21** to inhibit intracellular E2 formation was evaluated. Incubation of T47-D cells with the compound in the presence of labeled E1 resulted in a strong reduction of E2 formation within the cells (IC_50_ = 245 nM).

### 5) Binding mode analysis and SAR

In order to rationalize the influence of the different bridges in this new set of 17β-HSD1 inhibitors, and to investigate their potential binding modes, all tested compounds were docked in the pharmacophore model.

**Figure 9:**
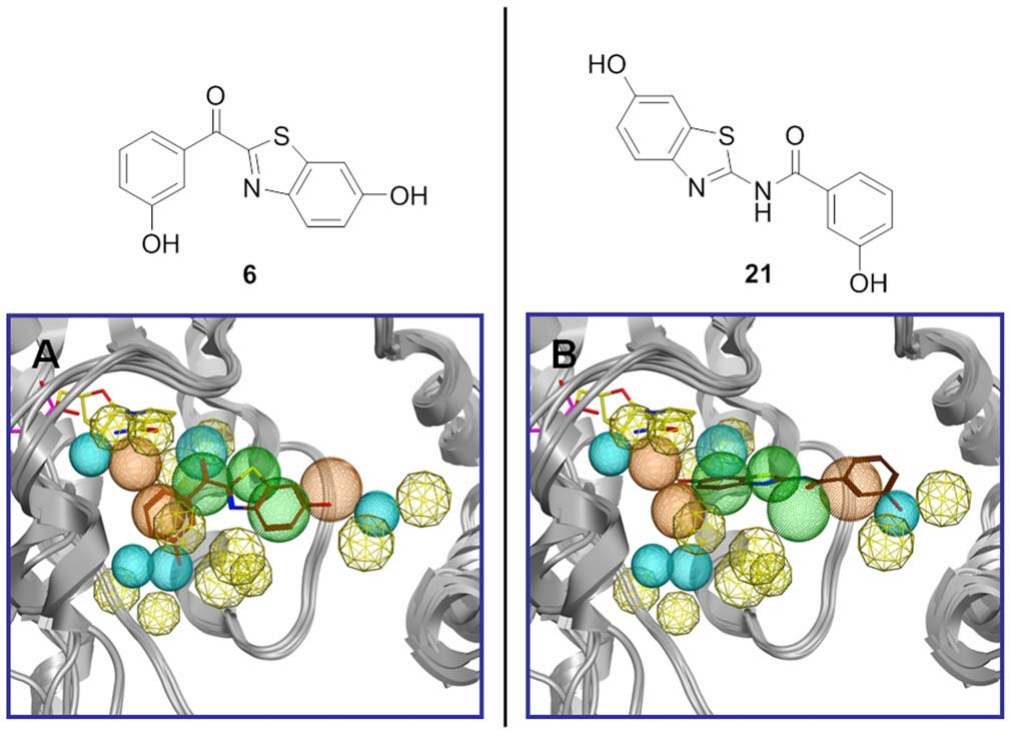
Pharmacophoric features exploited by 6 and 21. Six for compound **6** (showed in A) and five for compound **21** (showed in B).

The carbonyl compound **6** matches six pharmacophore features (**HY2-HY5**, **AD2** and **D4**), but it does not exploit the feature **HY1**, which corresponds to the steroidal A-ring moiety (see Figure 9A).

The retroamide **21** was found in a different orientation in the pharmacophore with respect to **6**, without occupying the feature **HY6**. The compound may adopt two preferred isomeric forms (*cis* and *trans*) which cannot be separated at room temperature because they interconvert readily. The energetically more favorable [74] linear (*trans*) isomer exploits the pharmacophoric features **HY1-HY4** and **AD1**. In addition, the hydroxy group of the benzothiazole moiety is situated in close proximity to the aromatic features **HY5** and **HY6**, as depicted in Figure 9B. The *cis* isomer, on the other hand, cannot match the required six features. Binding of **21** in the *trans*-form may thus be assumed. Interestingly, the retro amide bridge exploits the pharmacophoric feature **HY2**, which in the case of compound **6** is exploited by the benzothiazole phenyl ring. These data suggest very different binding modes for the two compounds.

To better understand the favourable interactions established by compounds **6** and **21** in the five different crystal structures used to build up the pharmacophore model, a thorough analysis of the respective surrounding residues was performed. For this purpose, also the flexible amino acid residues, formerly excluded from the pharmacophore generation process, were considered. The cocrystallized steroidal ligands were replaced by either **6** or **21** (via the pharmacophore) and the resultant complexes were optimized with the ligX module of MOE. This module optimizes the protein-ligand complex by first adjusting the protonation state of the residues, tethering the active site heavy atoms and, finally, energy minimizing the complex. The results highlighting the interactions between our 17β-HSD1 inhibitors and the five crystal structures are shown in Table 2.

**Table 2:** Interactions found in the complexes between 6, 21 and the five 17β-HSD1 crystal structures used to build up the pharmacophore, respectively.

Compound	interactions	amino acid residues	1equ	1i5r	3hb5	1a27	1dht
**6**	**H**	Ser142 (donor)		3.31	3.38	3.70	3.26
		Asn152 (acceptor)	2.53^a^	2.45	2.56		2.97
		Asn152 (donor)				2.49	2.55
		Tyr155 (donor)	3.90		3.80	3.71	
		His221 (d donor)	3.13	3.33			
	**π**	Tyr 155	6.71	3.98	3.82	4.06	4.16
		Arg258	4.89				
**21**	**H**	Tyr155 (acceptor)	2.91	3.13	3.03	2.75	3.03
		Tyr218 (donor)		3.82			
		His221 (donor)	2.47	2.45		2.65	2.51
		Glu282 (acceptor)	2.43		2.52		2.43
	**π**	Arg258	4.34				

a Distance (Å) between the heteroatoms for H-bonds (H) and between centroids or centroid and cation for π-interactions (π).

For compound **6** five hydrogen bond interactions could be observed: between its *meta* hydroxy group of the phenyl ring and Asn152 (d_O-O_ = 2.45–2.97 Å and d_O-N_ = 2.49–2.55 Å), between the carbonyl oxygen of the bridge and both Ser142 and Tyr155 (d_O-O_ = 3.26–3.70 Å and d_O-O_ = 3.71–3.90 Å), and between the hydroxy group of the benzothiazol and His221 (d_O-N_ = 3.13–3.33 Å). In addition, a cation-π interaction between the phenyl ring of benzothiazole and Arg258 (d_N-centroid_ = 4.89 Å) as well as a π-π interaction between the phenyl ring and Tyr155 (d_centroid-centroid_ = 3.98–6.71 Å) were found.

For compound **21** also five hydrogen bond interactions were identified: between the *meta* hydroxy group of the phenyl ring and both His221 and Glu282 (d_O-N_ = 2.45–2.65 Å and d_O-O_ = 2.43–2.52 Å, respectively), between the carbonyl oxygen of the amide bridge and Tyr218 (d_O-O_ 3.82 Å), and between the hydroxy group of the benzothiazole and Tyr155 (d_O-O_ = 2.75–3.13 Å). Again, a cation-π interaction between the phenyl ring and Arg258 was found (d_N-centroid_ = 4.34 Å).

It is noteworthy that only the interactions with Tyr155 (π-π stacking for **6** and hydrogen bond for **21**) could be observed for all five crystal structure-inhibitor complexes optimized with ligX, whereas all the other were present depending on which crystal structure was used. This further substantiated the importance of Tyr155 for the stabilization of a ligand.

**Figure 10:**
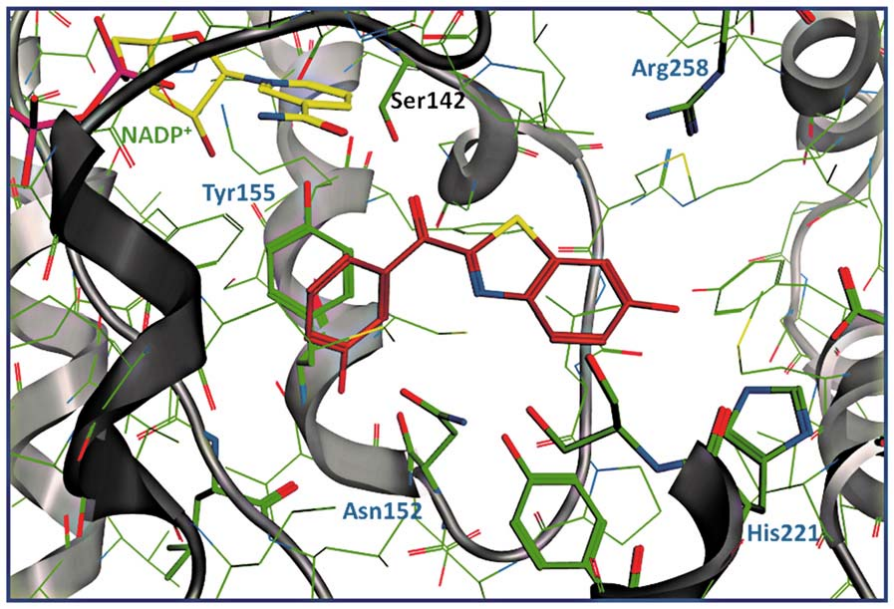
Pharmacophore derived complex between 17β-HSD1 (PDB-ID: 1equ) and compound 6 (dark orange). NADP^+^ (green), interacting residues (blue), potential interacting residues (black) and ribbon rendered tertiary structure of the active site are shown.

**Figure 11:**
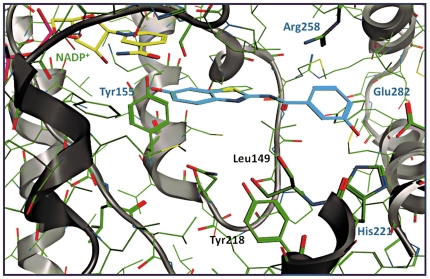
Pharmacophore derived complex between 17β-HSD1 (PDB-ID: 1equ) and compound 21 (magenta). NADP^+^ (green), interacting residues (blue), potential interacting residues (black) and ribbon rendered tertiary structure of the active site are shown.

The differences in inhibitory activity between **6** (IC_50_ = 44 nM) and **21** (IC_50_ = 243 nM) could not be thoroughly explained by considering only the hydrogen bonds, since only in 3hb5 and 1a27 more interactions were found for **6** compared to **21**. On the contrary, the π-π stacking interaction of **6** with Tyr155, missing for **21**, as well as the cation-π (charge transfer) interaction between Arg258 and the phenyl ring of benzothiazole seem to be particularly important for the 17β-HSD1 inhibition. Compound **21** was found to be involved only in π-stacking with Arg258, and no H-bond interactions with Ser142 were found in any of the complexes (exemplificative shown for 1equ, Figures 10 and 11).

In addition, the interactions between the hydroxy group in HY1 of compound **6** and His221, as found in different crystal structures (1equ and 1i5r), is in agreement with the postulated binding mode.

Summarizing, the two best inhibitors (**6** and **21**) here described bind very differently: they are flipped horizontally by 180° and the planes of their benzothiazole moieties form an angle of 90°.

## Discussion

The inhibitor design concept of the present study triggered the synthesis of compounds **6** and **21** as promising new 17β-HSD1 inhibitors by optimizing a novel, *in silico* identified, core scaffold (**5**).

The classical medicinal chemistry approach of rigidification was successfully applied to compound **5** and led to the discovery of the highly potent benzothiazole **6**. The introduction of the aromatic benzothiazole freezes the position of hydroxy group in an ideal position to establish an H-bond with H221. In addition, this aromatic benzothiazole can undergo a cation-π interaction with Arg258, explaining the high gain in potency of **6** compared to **5**.

In the optimization process the carbonyl bridge of **6** was varied using several linkers with different lengths, geometries and H-bonding properties. From the biological results as well as from the performed *in silico* studies it became apparent, that the 17β-HSD1 inhibitory activity is highly influenced by the nature of the linker: the comparison of inactive compounds showing a tetrahedral bridge geometry (**7, 8, 9, 10, 22**) with the active, planar carbonyl (**6**) and amide derivatives (**18** and **21**) led us to conclude that a flat geometry of the linker is required for activity. The fact that the retroamide **21** is five times more active than the amide **18** can be explained by a steric clash observed between the carbonyl of amide bridge and Leu149. Furthermore, the carbonyl group of **21** was found to establish an H-bond interaction with Tyr218 which is not possible for **18**.

Comparing the binding modes of **6** and **21**, it becomes apparent that the hydroxyphenyl moieties of the two compounds do not interact with the same area of the enzyme. In the case of compound **6**, **HY5** and **D4** are plausible features covered by the hydroxyphenyl moiety. The *meta*-hydroxyphenyl moiety of **21**, on the other hand, exploits **HY1** and **AD1**. The difference in activity between **6** and **21** is in agreement with the number of features covered by each compound (6 versus 5).

It is striking that the newly discovered class of benzothiazole derivatives shows structural characteristics which are similar to those of other classes of 17β-HSD1 inhibitors: two phenolic hydroxy-groups separated by a rather unpolar scaffold structure [48], [52], [54]. The necessity for the lipophilicity of the scaffold is reflected by the gain in potency observed with the thiourea (**24**: 62% inhibition at 1 µM) compared to the less lipophilic urea (**23**: no inhibition at 1 µM). The analysis of the amino acid residues which surround compound **6** in its pharmacophore binding pose indicates that two hydrogen bonds with Asn152 and one π-π interaction with Tyr155 are established. Recently published docking studies suggest similar interactions for bicyclic substituted hydroxyphenylmethanones [58]. Interestingly, there is a decrease of activity in both compound classes when the hydroxy group is shifted from the *meta*- to the *para* position. This similarity in SAR supports the hypothesis that the hydroxyphenyl moieties of both compound classes bind in the same area of the enzyme.

In order to evaluate the protein-ligand interactions, the ligands of the different X-ray structures studied were replaced by compounds **6** and **21** according to their pharmacophoric binding modes and the interactions between the inhibitors **6** and **21** and each of the crystal structures were examined. The maximum number of interactions was observed with the crystal structure 1equ, originally containing the inhibitor equiline. The reason for this is the residue Arg258 which protrudes into the active site in case of 1equ. The importance of this amino acid residue was already postulated by Alho-Richmond et al. [75], who proposed to target it in the inhibitor design process.

The biological assays employed for the evaluation of inhibitory potency towards 17β-HSD1 and 2 use well established conditions [59]. In the 17β-HSD1 assay, NADH rather than NADPH is used as cosubstrate. Substrate concentrations are adjusted to the corresponding K_m_-values which are reported in the literature [76]–[77] and confirmed by own experiments (data not given). Using NADH instead of the more expensive NADPH was found to give comparable results, as mentioned above (biological assays).

The selectivity against 17β-HSD2 should be achieved to mainly avoid systemic effects: This enzyme is downregulated in EDD tissues but is nevertheless present in several organs (i.e. liver, small intestine, bones). However, it is difficult to estimate how high the SF should be to minimize potential side effects due to the lack of respective *in vivo* data. For our drug development program, an SF of approximately 20 is considered sufficient to justify further biological evaluation. In this study the retroamide **21** is the most 17β-HSD2 selective compound identified. It is striking that the amide **18** shows a complete loss in selectivity against 17β-HSD2. As no 3D-structure of this enzyme is available, an interpretation of this result at protein level is not possible. The data indicate that the orientation of the amide group is an important feature to gain activity for 17β-HSD1 and selectivity against 17β-HSD2.

Affinity of the compounds to the ERs would counteract the therapeutic concept of mainly local action, no matter whether an agonistic or antagonistic effect is exerted. Basically, a possible estrogenic activity may be assessed using an estrogen-sensitive cell proliferation assay [59]. This rather laborious procedure is envisaged for a later stage of the drug optimization process. Earlier, we have found a good correlation between low RBA (relative binding affinity) and lack of ER-mediated cell proliferation [59]. We therefore used a different approach to quickly evaluate possible interference with the ERs, namely the determination of RBA values, or, more precisely, RBA intervals: For straightforward estimation of binding affinities, the range within which the RBA-value of a given compound is located was determined rather than the RBA-value itself. This approach should not be considered as a replacement for a proliferation assay but as a means to accelerate early stage drug design. Compounds exhibiting RBA values of less than 0.1% (RBA(E2) = 100%) were considered selective enough for potential *in vivo* application. This assumption is based on the comparison of the compound's binding affinity with that of E1. E1 itself is a ligand of the ERs with an RBA of about 10% [78]–[79]. As E1 is present in the diseased tissues, it competes with the inhibitor for binding to the ERs. Due to its low RBA value (less than 0.1%), **21** should be displaced by E1 from the ER binding site and is thus unlikely to exert an ER mediated effect *in vivo*. On the contrary, compound **6** shows enhanced affinity to the ERs. This data, however, does not allow to conclude whether the compound acts as an agonist or an antagonist – but this is not relevant in terms of the pursued therapeutic concept which aims at excluding systemic effects as far as possible. Of course, an agonistic effect would be negative for the treatment of estrogen-dependent diseases and can obviously not be tolerated. An antagonistic mode of action, on the other hand, will lead to systemic effects in other, healthy steroidogenic tissues, undoing the concept of local action. Therefore, we focused on the discovery of compounds without low affinities to the ERs without regarding agonistic or antagonistic action.

In the present study two new classes of 17β-HSD1 inhibitors were identified. As no X-ray structure of the target enzyme complexed with nonsteroidal compounds exists, a pharmacophoric approach was followed which combines three-dimensional information of the protein and complexed steroidal inhibitors with the structure analysis of nonsteroidal inhibitors. Virtual screening using the derived pharmacophore model resulted in the identification of the fairly active hit compound **5** which was the basis for structural modifications leading to benzothiazole-based compounds with favourable biological activities. Correlating their inhibitory potencies with the pharmacophore model gave information about probable binding modes.

In this study, the benzothiazole **6** is the most active compound in terms of 17β-HSD1 inhibition (IC_50_ = 44 nM). It is selective against 17β-HSD2 (SF = 24) but shows pronounced affinity to the ERs (RBA<10% for ERα and RBA<1% for ERβ). Compound **21** on the other hand showed medium inhibitory activity at the target enzyme (IC_50_ = 243 nM) as well as selectivity not only against 17β-HSD2 (SF = 38) but also against the ERs (RBA<0.1% for ERα and RBA<0.001% for ERβ). Furthermore, **21** strongly inhibits the intracellular formation of E2 in T47-D cells (IC_50_ = 245 nM). Further optimizations of these first benzothiazole-type 17β-HSD1 inhibitors are underway in order to develop potential candidates for *in vivo* application.

## Supporting Information

Figure S1 **Compound 6 (dark orange) mapped to the pharmacophore model and overlaid with equiline (yellow).**
(TIF)

Table S1 17β-HSD1 inhibitory activity for compounds 5 and 6–25. ^a^ Human placenta, cytosolic fraction, substrate [^3^H]E1 + E1 [500 nM], cofactor NADH [500 µM]. ^b^ Mean values of three determinations, standard deviation less than 10%. ^c^ nd: not determined. ^d^ ni: no inhibition.
(DOC)

Table S2 Geometrical properties of the extended pharmacophore model.
(DOC)

Table S3 Small *in house* library.
(DOC)

Table S4 Influence of bridge and hydroxy group on the inhibition of human 17β-HSD1 and 17β-HSD2. ^a^ Human placenta, cytosolic fraction, substrate [^3^H]E1 + E1 [500 nM], cofactor NADH [500 µM]. ^b^ Human placenta, microsomal fraction, substrate [^3^H]E2 + E2 [500 nM], cofactor NAD^+^ [1500 µM]. ^c^ Mean values of three determinations, standard deviation less than 10%. ^d^ Selectivity factor = IC_50_ (17β-HSD2)/IC_50_(17β-HSD1).
(DOC)

File S1 Supporting Information.
(DOC)
