# Supplementary figures and images for: Hydroxybenzothiazoles as New Nonsteroidal Inhibitors of 17β-Hydroxysteroid Dehydrogenase Type 1 (17β-HSD1)
Source: PLoS One. 2012 Jan 5;7(1):e29252. doi: 10.1371/journal.pone.0029252 (PMC3252304; doi:10.1371/journal.pone.0029252)

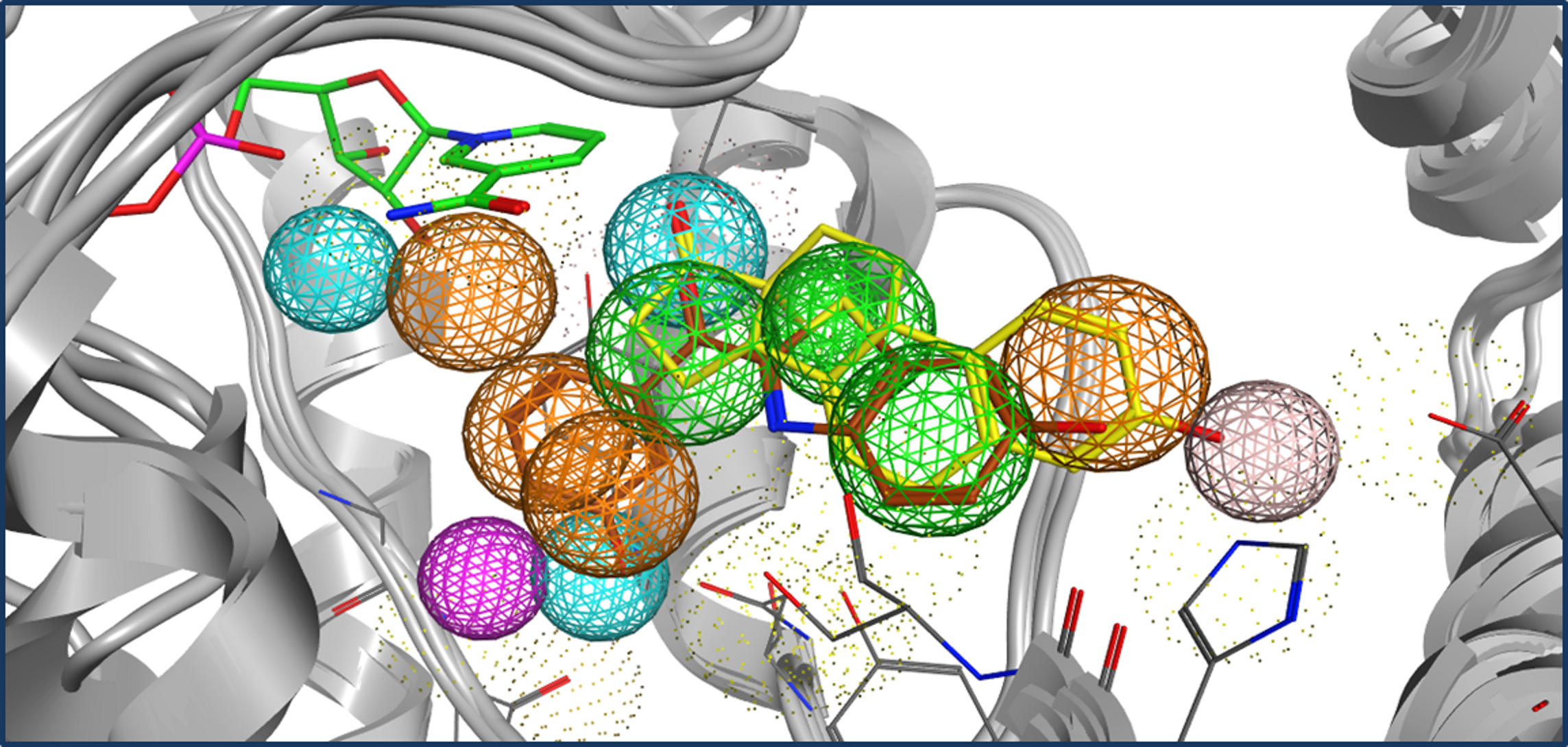

Supplement: Figure S1 — Compound 6 (dark orange) mapped to the pharmacophore model and overlaid with equiline (yellow). (TIF) [file pone.0029252.s001.tif]
