# Supplementary material for: Hydroxybenzothiazoles as New Nonsteroidal Inhibitors of 17β-Hydroxysteroid Dehydrogenase Type 1 (17β-HSD1)
Source: PLoS One. 2012 Jan 5;7(1):e29252. doi: 10.1371/journal.pone.0029252 (PMC3252304; doi:10.1371/journal.pone.0029252)
Supplement: Table S1 — 17β-HSD1 inhibitory activity for compounds 5 and 6–25. a Human placenta, cytosolic fraction, substrate [3H]E1 + E1 [500 nM], cofactor NADH [500 µM]. b Mean values of three determinations, standard deviation less than 10%. c nd: not determined. d ni: no inhibition. (DOC) [file pone.0029252.s002.doc]

**Table S1. 17β-HSD1 inhibitory activity for compounds 5 and 6-25.**


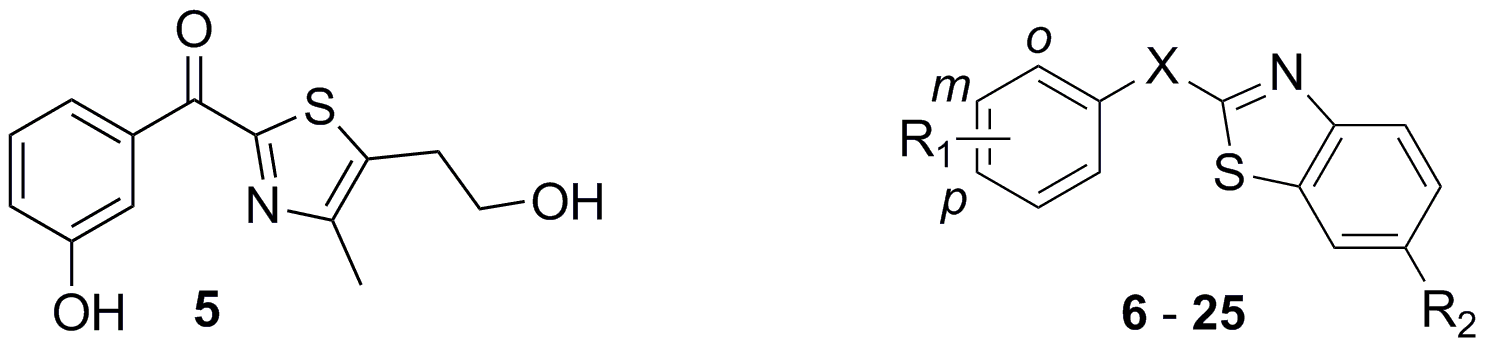


| **Compound** | **X** | **R_1_** | **R_2_** | **17β-HSD1**^a^ | |
| --- | --- | --- | --- | --- | --- |
|  |  |  |  | **% inhib. (1µM)** | **IC_50_**^b^ **(nM)** |
| **5** |  |  |  | 34 | nd |
| **6** | CO | *m*-OH | OH | 91 | 44 |
| **7** | CHOH | *m*-OH | OH | 28 | nd |
| **8** | CHOCH_3_ | *m*-OH | OH | 13 | nd |
| **9** | CHSCH_3_ | *p*-OH | OH | ni | nd |
| **10** | CH_2_ | *p*-OH | OCH_3_ | ni | nd |
| **11** | CH_2_ | *p*-OH | OH | 24 | nd |
| **12** | CO | *m*-OH | H | 75 | 365 |
| **6i** | CO | *m*-OCH_3_ | OCH_3_ | ni. | nd |
| **13** | CO | *p*-OCH_3_ | OH | 27 | nd |
| **14** | CO | *p*-OH | OH | 85 | 243 |
| **15** | NHCO | *o*-OH | OCH_3_ | ni | nd |
| **16** | NHCO | *o*-OH | OH | ni | nd |
| **17** | NHCO | *m*-OH | OCH_3_ | 31 | nd |
| **18** | NHCO | *m*-OH | OH | 40 | nd |
| **19** | CONH | *p*-OCH_3_ | OH | ni. | nd |
| **20** | CONH | *p*-OH | OH | 39 | nd |
| **21** | CONH | *m*-OH | OH | 83 | 243 |
| **22** | SO_2_NH | *m*-OH | OH | ni | nd |
| **23** | NHCONH | *m*-OH | OH | ni. | nd |
| **24** | NHCSNH | *m*-OH | OH | 62 | nd |
| **25** | CH_2_CONH | *m*-OH | OH | ni | nd |

^a^ Human placenta, cytosolic fraction, substrate [^3^H]E1 + E1 [500 nM], cofactor NADH [500 μM]. ^b^ Mean values of three determinations, standard deviation less than 10 %. ^c^ nd: not determined. ^d^ ni: no inhibition.
