# Supplementary material for: Hydroxybenzothiazoles as New Nonsteroidal Inhibitors of 17β-Hydroxysteroid Dehydrogenase Type 1 (17β-HSD1)
Source: PLoS One. 2012 Jan 5;7(1):e29252. doi: 10.1371/journal.pone.0029252 (PMC3252304; doi:10.1371/journal.pone.0029252)
Supplement: Table S2 — Geometrical properties of the extended pharmacophore model. (DOC) [file pone.0029252.s003.doc]

**Table S2.** **Geometrical properties of the extended pharmacophore model.**

| **distances (Å)** | | **angles (°)** | | **radii of spheres (Å)** | |
| --- | --- | --- | --- | --- | --- |
| **A1a**-**D1b** | 4.0 | **A6b**-**HY6**-**D6a** | ~42 | **A1a** | 1.5 |
| **A3a**-**D4b** | 2.8 | **AD1**-**HY1**-**HY2** | ~129 | **A3a** | 1.1 |
| **A6b**-**D6a** | 2.2 | **AD2**-**AD2a**-**AD2b** | ~67 | **A6b** | 1.4 |
| **AD1**-**A1a** | 2.8 | **AD2**-**AD2b**-**AD2a** | ~59 | **AD1** | 1.2 |
| **AD1**-**D1b** | 2.6 | **AD3**-**A3a**-**D4b** | ~82 | **AD2** | 1.5 |
| **AD1**-**HY1** | 2.8 | **AD5b**-**HY2**-**AD5a** | ~54 | **AD2a** | 1.4 |
| **AD2a**-**AD2b** | 2.6 | **D4**-**A3a**-**D4b** | ~54 | **AD2b** | 1.5 |
| **AD2**-**AD2a** | 2.7 | **D4b**-**AD3**-**A3a** | ~50 | **AD3** | 1.2 |
| **AD2**-**AD2b** | 2.9 | **D4**-**D4a**-**D4b** | ~41 | **AD5a** | 1.2 |
| **AD3**-**D4** | 2.2 | **D4**-**D4b**-**A3a** | ~77 | **AD5b** | 1.5 |
| **AD5a**-**AD5b** | 3.3 | **D4**-**D4b**-**D4a** | ~43 | **D1b** | 1.3 |
| **AD5a**-**D4a** | 3.9 | **D6a**-**A6b**-**HY6** | ~78 | **D4** | 1.3 |
| **D4a**-**D4b** | 4.7 | **D7**-**AD3**-**D4** | ~115 | **D4a** | 1.7 |
| **D4**-**D4a** | 3.2 | **HY1**-**A1a**-**D1b** | ~75 | **D4b** | 1.3 |
| **D4**-**D4b** | 3.0 | **HY1**-**AD1**-**HY2** | ~27 | **D6a** | 1.4 |
| **D7**-**A6b** | 2.8 | **HY1**-**D1b**-**A1a** | ~55 | **D7** | 1.2 |
| **HY1**-**HY2** | 3.0 | **HY1**-**HY2**-**A1a** | ~52 | **HY1** | 1.7 |
| **HY2**-**AD5a** | 4.7 | **HY1**-**HY3**-**HY2** | ~41 | **HY2** | 1.7 |
| **HY2**-**AD5b** | 5.8 | **HY2**-**AD5a**-**AD5b** | ~75 | **HY3** | 1.5 |
| **HY2**-**D4a** | 3.9 | **HY2**-**AD5b**-**AD5a** | ~51 | **HY4** | 1.7 |
| **HY2**-**HY3** | 2.7 | **HY2**-**HY3**-**HY4** | ~115 | **HY5** | 1.5 |
| **HY3**-**HY4** | 2.9 | **HY3**-**AD2**-**HY4** | ~47 | **HY6** | 1.5 |
| **HY4**-**AD2** | 2.6 | **HY3**-**HY2**-**HY4** | ~34 |  |  |
| **HY4**-**HY5** | 3.0 | **HY3**-**HY4**-**HY2** | ~31 |  |  |
| **HY4**-**HY6** | 4.1 | **HY4**-**AD2**-**HY5** | ~40 |  |  |
| **HY5**-**AD3** | 3.5 | **HY4**-**AD3**-**D4** | ~60 |  |  |
| **HY5**-**D4** | 3.7 | **HY4**-**D4**-**AD3** | ~102 |  |  |
| **HY5**-**P5** | 2.1 | **HY4**-**D7**-**AD2** | ~23 |  |  |
| **HY6**-**A6a** | 3.2 | **HY4**-**D7**-**AD3** | ~56 |  |  |
| **HY6**-**A6b** | 2.9 | **HY4**-**HY3**-**AD2** | ~40 |  |  |
| **HY6**-**D7** | 2.5 | **HY4**-**HY5**-**AD3** | ~146 |  |  |
| **P5**-**HY4** | 2.9 | **HY4**-**HY5**-**D4** | ~110 |  |  |
|  |  | **HY4**-**HY5**-**P5** | ~70 |  |  |
|  |  | **HY4**-**HY6**-**AD2** | ~37 |  |  |
|  |  | **HY4**-**HY6**-**D7** | ~167 |  |  |
|  |  | **HY4**-**HY6**-**HY5** | ~45 |  |  |
|  |  | **HY4**-**P5**-**HY5** | ~66 |  |  |
