# Supplementary material for: Hydroxybenzothiazoles as New Nonsteroidal Inhibitors of 17β-Hydroxysteroid Dehydrogenase Type 1 (17β-HSD1)
Source: PLoS One. 2012 Jan 5;7(1):e29252. doi: 10.1371/journal.pone.0029252 (PMC3252304; doi:10.1371/journal.pone.0029252)
Supplement: Table S3 — Small in house library. (DOC) [file pone.0029252.s004.doc]

**Table S3.** **Small *in house* library.**

| **Compound** | **Y** | **Z** | **X** | **R_1_** | **R_2_** | **R_3_** | **R_4_** |
| --- | --- | --- | --- | --- | --- | --- | --- |
| I | CH | CH | NH | 2-OCH_3_ | 5-CF_3_ | CH_3_ | H |
| II | CH | CH | NH | 2-OCH_3_ | 5-CF_3_ | CH_3_ | CH_3_ |
| III | CH | CH | NH | 2-OCH_3_ | 5-CF_3_ | CH_2_CH_2_CH_3_ | H |
| IV | CH | CH | NH | 2-OCH_3_ | 5-CF_3_ | CH_3_ | COOCH_2_CH_3_ |
| V | CH | CH | NH | 2-OCH_3_ | 5-CF_3_ | CH_3_ | COCH_3_ |
| VI | CH | CH | NH | 2-OCH_3_ | 5-CF_3_ | H | CH_2_CH_2_OH |
| VII | CH | CH | NH | 2-OCH_3_ | 5-CF_3_ | CH(CH_2_CH_3_)(OCH_2_CH_3_) | H |
| VIII | CH | CH | CH_2_ | 2-OCH_3_ | 4-CF_3_ | CH_3_ | CH_2_CH_2_OH |
| IX | CH | CH | CH_2_ | 2-OCH_3_ | 5-CF_3_ | CH_3_ | CH_2_CH_2_OH |
| X | CH | CH | CH_2_ | 3-OH | H | CH_3_ | CHCH_2_ |
| XI | CH | CH | CH_2_ | 3-OCH_3_ | H | CH_3_ | CH_2_CH_2_OH |
| XII | CH | CH | CH_2_ | 4-OCH_3_ | H | CH_3_ | CH_2_CH_2_OH |
| XIII | CH | CH | CH_2_ | 4-OH | H | CH_3_ | CHCH_2_ |
| XIV | CH | CH | CHOH | 3-OH | H | CH_3_ | CH_2_CH_2_OH |
| XV | CH | CH | CHOH | 2-OCH_3_ | 4-CF_3_ | CH_3_ | CH_2_CH_2_OH |
| XVI | CH | CH | CHOH | 2-OCH_3_ | 5-CF_3_ | CH_3_ | CH_2_CH_2_OH |
| XVII | CH | CH | CHOH | 4-OH | H | CH_3_ | CH_2_CH_2_OH |
| XVIII | CH | CH | CHOH | 3-OCH_3_ | H | CH_3_ | CH_2_CH_2_OH |
| XIX | CH | CH | CHOH | 4-OCH_3_ | H | CH_3_ | CH_2_CH_2_OH |
| XX | CH | CH | CO | 3-OCH_3_ | H | CH_3_ | CH_2_CH_2_OH |
| XXI | CH | CH | CO | 3-OH | H | CH_3_ | CH_2_CH_2_OH |
| XXII | CH | CH | CO | 4-OCH_3_ | H | CH_3_ | CH_2_CH_2_OH |
| XXIII | CH | CH | CO | 4-OH | H | CH_3_ | CH_2_CH_2_OH |
| XXIV | CH | CH | CH_2_NH | 2-OCH_3_ | 4-CF_3_ | CH_3_ | CH_2_CH_2_OH |
| XXV | CH | CH | CH_2_NH | 2-OCH_3_ | 5-CF_3_ | CH_3_ | CH_2_CH_2_OCOCH_3_ |
| XXVI | CH | CH | CONH | 2-OCH_3_ | 5-CF_3_ | CH_3_ | CH_2_CH_2_OCOCH_3_ |
| XXVII | CH | CH | CONH | 2-OCH_3_ | 5-CF_3_ | CH_3_ | CH_2_CH_2_OH |
| XXVIII | CH | CH | CONH | 2-OCH_3_ | 4-CF_3_ | CH_3_ | CH_2_CH_2_OH |
| XXIX | N | CH | CH_2_ | H | H | CH_3_ | CH_2_CH_2_OH |
| XXX | N | CH | CONH | 2-OCH_3_ | H | CH_3_ | CH_2_CH_2_OH |
| XXXI | N | CH | CONH | 2-OCH_3_ | H | CH_3_ | CH_2_CH_2_OCH_3_ |
| XXXII | N | CH | CONH | 2-OCH_3_ | H | CH_3_ | CH_2_CH_2_OCOCH_3_ |
| XXXIII | N | CH | CONH | 2-OCH_3_ | H | CH_3_ | CH_2_CH_2_OCOCH_3_ |
| XXXIV | N | N | CONH | 2-OCH_3_ | 4-CH_3_ | CH_3_ | CH_2_CH_2_OH |
| XXXV | CH | CH | CH_2_ | H | H | CH_3_ | CH_2_CH_2_OH |
| XXXVI | CH | CH | CH_2_ | 2-OCH_3_ | 5-CF_3_ | CH_3_ | CH_2_CH_2_OH |
| XXXVII | N | N | CH_2_ | 2-OH | 4-CH_3_ | CH_3_ | CH_2_CH_2_OH |
