# Supplementary material for: Hydroxybenzothiazoles as New Nonsteroidal Inhibitors of 17β-Hydroxysteroid Dehydrogenase Type 1 (17β-HSD1)
Source: PLoS One. 2012 Jan 5;7(1):e29252. doi: 10.1371/journal.pone.0029252 (PMC3252304; doi:10.1371/journal.pone.0029252)
Supplement: Table S4 — Influence of bridge and hydroxy group on the inhibition of human 17β-HSD1 and 17β-HSD2. a Human placenta, cytosolic fraction, substrate [3H]E1 + E1 [500 nM], cofactor NADH [500 µM]. b Human placenta, microsomal fraction, substrate [3H]E2 + E2 [500 nM], cofactor NAD+ [1500 µM]. c Mean values of three determinations, standard deviation less than 10%. d Selectivity factor = IC50 (17β-HSD2)/IC50(17β-HSD1). (DOC) [file pone.0029252.s005.doc]

**Table S4. Influence of bridge and hydroxy group on the inhibition of human 17β-HSD1 and 17β-HSD2.**


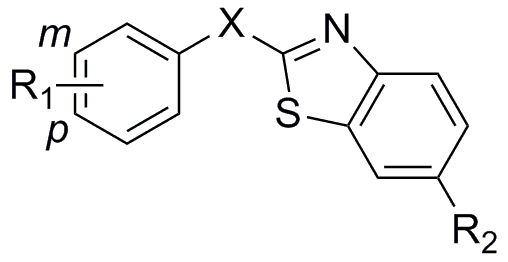


| **Compound** | **X** | **R_1_** | **R_2_** | **17β-HSD1^a^**  **[IC_50_ (nM)^c^]** | **17β-HSD2^b^**  **[IC_50_ (nM)^c^]** | **SF^d^** |
| --- | --- | --- | --- | --- | --- | --- |
| **6** | CO | *m*-OH | OH | 44 | 1035 | 24 |
| **12** | CO | *m*-OH | H | 365 | 1356 | 4 |
| **14** | CO | *p*-OH | OH | 243 | 2471 | 10 |
| **18** | NHCO | *m*-OH | OH | 1307 | 3813 | 3 |
| **21** | CONH | *m*-OH | OH | 243 | 9264 | 38 |

^a^ Human placenta, cytosolic fraction, substrate [^3^H]E1 + E1 [500 nM], cofactor NADH [500 μM]. ^b^ Human placenta, microsomal fraction, substrate [^3^H]E2 + E2 [500 nM], cofactor NAD^+^ [1500 μM]. ^c^ Mean values of three determinations, standard deviation less than 10 %. ^d^ Selectivity factor = IC_50_ (17β-HSD2)/IC_50_(17β-HSD1).
