# Supplementary material for: Hydroxybenzothiazoles as New Nonsteroidal Inhibitors of 17β-Hydroxysteroid Dehydrogenase Type 1 (17β-HSD1)
Source: PLoS One. 2012 Jan 5;7(1):e29252. doi: 10.1371/journal.pone.0029252 (PMC3252304; doi:10.1371/journal.pone.0029252)
Supplement: File S1 — Supporting Information. (DOC) [file pone.0029252.s006.doc]

**Supporting Information**

**Hydroxybenzothiazoles as New Nonsteroidal Inhibitors of 17β-Hydroxysteroid Dehydrogenase Type 1 (17β-HSD1)**

Alessandro Spadaro^a,b^, Matthias Negri^c^, Sandrine Marchais-Oberwinkler^a^, Emmanuel Bey^b^, Martin Frotscher^a^

Author affiliation:

^a^ Pharmaceutical and Medicinal Chemistry, Saarland University, Saarbrücken, Germany;

^b^ ElexoPharm GmbH, Saarbrücken, Germany;

^c^ Helmholtz Institute for Pharmaceutical Research Saarland (HIPS), Saarbrücken, Germany.

Corresponding author:

Dr. Martin Frotscher. Pharmaceutical and Medicinal Chemistry, Saarland University, Campus C23, 66123 Saarbrücken, Germany; phone: +(49) 681-30270330; e-mail: m.frotscher@mx.uni-saarland.de; Homepage: [http://www.PharmMedChem.de](http://www.PharmMedChem.de/)

**Experimental section**

**Chemical Methods.** Chemical names follow IUPAC nomenclature. Starting materials were purchased from Acros, Aldrich, Alfa Aesar, Maybridge, Merck or Fluka and were used without purification.

Column chromatography (CC) was performed on silica gel (70-200 µm) coated with silica, preparative thin layer chromatography (TLC) on 1 mm SIL G-100 UV_254_ glass plates (Macherey-Nagel) and reaction progress was monitored by TLC on Alugram SIL G UV_254_ (Macherey-Nagel).

^1^H-NMR and ^13^C-NMR spectra were measured on a Bruker AM500 spectrometer (500 MHz) at 300 K. Chemical shifts are reported in δ (parts per million: ppm), by reference to the hydrogenated residues of deuteriated solvent as internal standard CDCl_3_: δ = 7.24 ppm (^1^H-NMR) and δ = 77 ppm (^13^C-NMR), CD_3_OD: δ = 3.32 ppm (^1^H-NMR) and δ = 49.05 ppm (^13^C-NMR), CD_3_COCD_3_: δ = 2.05 ppm (^1^H-NMR) and δ = 29.9 ppm (^13^C-NMR) and CD_3_SOCD_3_: δ = 2.50 ppm (^1^H-NMR) and δ = 39.43 ppm (^13^C-NMR). Signals are described as s, d, t, dd, ddd, m, dt, q for singlet, doublet, triplet, doublet of doublets, doublet of doublets of doublets, multiplet, doublet of triplets and quadruplet respectively. All coupling constants (*J*) are given in hertz (Hz).

Mass spectra (ESI) were recorded on a TSQ Quantum (Thermo Finnigan) instrument.

Tested compounds are > 95 % chemical purity as measured by HPLC.

The following compounds were prepared according to previously described procedures: 3-(*tert*-butyldimethylsilanyloxy)benzaldehyde (**5iiib**) [1], 6-methoxy-1,3-benzothiazole (**6iii**) [2]**,** (6-hydroxy-1,3-benzothiazol-2-yl)(3-hydroxyphenyl)methanone (**6**) [3], (6-methoxy-1,3-benzothiazol-2-yl)(4-methoxyphenyl)methanone (**13i**) [3], 1,3-benzothiazol-2-yl(3-hydroxyphenyl)methanone (**12**) [4], (6-hydroxy-1,3-benzothiazol-2-yl)(4-methoxyphenyl)methanone (**13**) [3], (6-hydroxy-1,3-benzothiazol-2-yl)(4-hydroxyphenyl)methanone (**14**) [3], 4-methoxy-*N*-(6-methoxy-1,3-benzothiazol-2-yl)benzamide (**19i**) [5], 3-methoxy-*N*-(6-methoxy-1,3-benzothiazol-2-yl)benzamide (**21i**) [5].

**Method A, general procedure for nucleophilic addition:** To a solution of thiazole derivative unsubstituted in position 2 (1 eq) in anhydrous THF a 2.5 M solution of *n-*BuLi (1 eq) in hexane was added dropwise at -78 °C (dry ice/acetone bath) under anhydrous conditions. The reaction mixture was stirred for 1 h at –78 °C to form the formyl anion in situ. Then a solution of electrophile (0.8 eq) in anhydrous THF was added dropwise at –78 °C and additionally stirred for 30 min. The temperature was then risen to –20 °C (NaCl/ice/dry ice/acetone bath) and the stirring was continued for 90 min. Saturated NH_4_Cl solution was added to quench the reaction, and the aqueous layer was extracted three times with ethyl acetate. The combined organic layers were washed with brine, dried over magnesium sulfate, filtered and concentrated to dryness. The product was purified by CC or recrystallisation.

**Method B, general procedure for nucleophilic substitution:** To a solution of 2-amino-6-methoxy-1,3-benzothiazole (1 eq) in dry pyridine the respective benzoyl chloride, isocyanate or isothiocianate (1 eq.) was added dropwise at room temperature. The reaction mixture was refluxed for 4 h. Water was added to quench the reaction, and the aqueous layer was extracted with ethyl acetate. The combined organic layers were washed with brine, dried over sodium sulfate, filtered, and concentrated to dryness. The product was purified by CC or recrystallisation.

**Method C, general procedure for oxidation:** A mixture of aliphatic alcohol (1 eq) and 2-iodoxybenzoic acid (2 eq) in anhydrous THF was stirred at 0 °C for 10 min. Then, the reaction mixture was heated to 60 °C for 18 h. After cooling to room temperature, saturated sodium thiosulfate solution was added to quench the reaction and the aqueous layer was extracted with ethyl acetate. The combined organic layers were washed with brine, dried over magnesium sulfate, filtered and concentrated to dryness. The product was purified by CC or recrystallisation.

**Method D, general procedure for ether cleavage:** To a solution of methoxybenzene derivative (1 eq) in dry dichloromethane at room temperature, boron trifluoride methyl sulfide complex in dichloromethane (1 M, 75 eq per methoxy function) was added dropwise. The reaction mixture was stirred for 20 h at room temperature under nitrogen atmosphere. Water was added to quench the reaction, and the aqueous layer was extracted with ethyl acetate. The combined organic layers were washed with brine, dried over magnesium sulfate, filtered and concentrated to dryness. The product was purified by CC, preparative TLC, preparative HPLC or recrystallisation, respectively.

**Method E, general procedure for ether cleavage:** A mixture of methoxybenzene derivative (1 eq) and pyridinium hydrochloride (50 eq per methoxy function) was heated to 220 °C for 18 h. After cooling to room temperature, water, 1 M HCl and ethyl acetate were added. The aqueous layer was separated and extracted with ethyl acetate. The combined organic layers were washed with brine, dried over sodium sulfate, filtered and concentrated to dryness. The product was purified by CC followed by preparative TLC or preparative HPLC, respectively.

**Method F, general procedure for ether cleavage:** To a solution of methoxybenzene derivative (1 eq) in anhydrous dichloromethane at -78 °C (dry ice/acetone bath), boron tribromide in dichloromethane (1 M, 5 eq per methoxy function) was added dropwise. The reaction mixture was stirred for 20 h at room temperature under nitrogen atmosphere. Water was added to quench the reaction, and the aqueous layer was extracted with ethyl acetate. The combined organic layers were washed with brine, dried over sodium sulfate, filtered and concentrated to dryness. The product was purified by CC followed by preparative TLC or preparative HPLC, respectively.

**General procedure for purification using preparative HPLC.** All declared final compounds were purified via an Agilent Technologies Series 1200-preparative HPLC using a linear gradient run (solvents: acetonitrile, water) from 20 % acetonitrile to 100 % in 36 min.

**5-[2-(*tert*-Butyldimethylsilanyloxy)-ethyl]-4-methylthiazole** (**5iiia**). To a solution of 5-(2-hydroxyethyl)-4-methyl-thiazole (8.0 g, 55.86 mmol) and *tert*-butyldimethylsilyl chloride (12.63 g, 83.79 mmol) in DMF, imidazole (6.46 g, 94.97 mmol) was added in portion for 1 hour at 0 °C. The reaction mixture was stirred for 6 h at room temperature. Water was added to quench the reaction and the aqueous solution was then extracted three times with ethyl acetate. The combined organic layers were washed with brine, dried over magnesium sulfate, filtered and concentrated to dryness. The product was used in the next synthetic step without purification; yield: quant. (14.38 g); ^1^H NMR (CDCl_3_): 0.06 (s, 6H), 0.80 (s, 9H), 2.32 (s, 3H), 2.88 (t, *J* = 6.0 Hz, 2H), 3.70 (t, *J* = 6.0 Hz, 2H), 8.47 (s, 1H); ^13^C NMR (CDCl_3_): -2.3, 15.3, 24.9, 32.0, 34.6, 62.2, 127.3, 149.2, 150.0;

**{5-[2-(*tert*-Butyldimethylsilanyloxy)ethyl]-4-methylthiazol-2-yl}-[3-(*tert*-butyldimethyl-silanyloxy)phenyl]methanol** (**5i**). The title compound was prepared by reaction of 5-[2-(*tert*-butyldimethylsilanyloxy)ethyl]-4-methylthiazole (**5iiia**) (5 g, 19.42 mmol), *n-*BuLi (2,5 M in hexane; 7.8 mL, 19.42 mmol) and 3-(*tert*-butyl-dimethyl-silanyloxy)-benzaldehyde (**5iiib**) (3.53 g, 14.94 mmol) according to method A. The product was purified by CC (hexane/ethyl acetate 80:20); yield: 64 % (4.06 g); ^1^H NMR (CDCl_3_): **-**0.03 (s, 6H), 0.18 (s, 6H), 0.84 (s, 9H), 0.97 (s, 9H), 2.30 (s, 3H), 2.86 (t, *J* = 6.5 Hz, 2H), 3.70 (t, *J* = 6.5 Hz, 2H), 5.89 (s, 1H), 6.77 (dd, *J* = 8.2 Hz, *J* = 2.5 Hz, 1H), 6.96 (s, 1H), 7.03-7.05 (m, 1H), 7.20 (t, *J* = 7.9 Hz, 1H); ^13^C NMR (CDCl_3_): -5.5, -4.4, 18.2, 18.2, 21.0, 25.7, 25.8, 30.0, 63.2, 73.2, 118.2, 119.4, 119.8, 129.3, 129.5, 143.2, 147.6, 155.8, 171.1;

**{5-[2-(*tert*-Butyldimethylsilanyloxy)ethyl]-4-methylthiazol-2-yl}-[3-(*tert*-butyldimethyl-silanyloxy)phenyl]-methanone** (**5i**). The title compound was prepared by reaction of {5-[2-(*tert*-butyldimethylsilanyloxy)ethyl]-4-methylthiazol-2-yl}-[3-(*tert*-butyldimethylsilanyloxy)-phenyl]methanol (**5ii**) (0.5 g, 1.01 mmol) and SIBX (0.57 g, 2.02 mmol) according to method C. The product was purified by CC (hexane/ethyl acetate 95:5); yield: 94 % (0.47 g); ^1^H NMR (CD_3_OD) 2.33 (s, 3H), 2.91 (t, *J* = 6.0 Hz, 2H), 3.68 (t, *J* = 6.0 Hz, 2H), 6.94 (d, *J* = 7.9 Hz, 1H), 7.20 (t, *J* = 8.0 Hz, 1H), 7.59 (s, 1H), 7.72 (d, *J* = 7.9 Hz, 1H); ^13^C NMR (CD_3_DO): 15.1, 30.3, 62.0, 117.3, 121.1, 122.8, 129.6, 136.7, 138.2, 152.0, 152.1, 157.1, 163.3;

**[5-(2-Hydroxyethyl)-4-methylthiazol-2-yl]-(3-hydroxyphenyl)methanone** (**5**). To a solution of {5-[2-(*tert*-butyldimethylsilanyloxy)ethyl]-4-methylthiazol-2-yl}-[3-(*tert*-butyldimethylsilanyloxy)phenyl]methanone (**5i**) (0.47 g, 0.96 mmol) in THF at 0 °C (dry ice) tetra-*n-*butylammonium fluoride (0.62 g, 2.38 mmol) in THF was added dropwise. The reaction mixture was stirred for 2 h at room temperature under nitrogen atmosphere. Water was added to quench the reaction, and the aqueous layer was extracted with ethyl acetate. The combined organic layers were washed with brine, dried over magnesium sulfate, filtered and concentrated to dryness. The product was purified by CC (hexane/ethyl acetate 50:50); yield: 80 % (0.20 g); ^1^H NMR (CD_3_OD): 2.33 (s, 3H), 2.91 (t, *J* = 6.4 Hz, 2H), 3.68 (t, *J* = 6.4 Hz, 2H), 6.94 (d, *J* = 7.9 Hz, 1H), 7.20 (t, *J* = 8.0 Hz, 1H), 7.59 (s, 1H), 7.72 (d, *J* = 7.9 Hz, 1H); ^13^C NMR (CD_3_DO): 15.1, 30.3, 62.0, 117.3, 121.1, 122.8, 129.6, 136.7, 138.2, 152.0, 152.1, 157.1, 163.3; MS (ESI): 264.0 (M+H)^+^;

**(6-Methoxy-1,3-benzothiazol-2-yl)(3-methoxyphenyl)methanol** (**6ii**)**.** The title compound was prepared by reaction of 6-methoxy-1,3-benzothiazole (**6iii**) (0.5 g, 3.03 mmol), *n-*BuLi (2,5 M in hexane; 1.2 mL, 3.03 mmol) and 3-methoxy-benzaldehyde (0.27 mL, 2.32 mmol) according to method A. The product was purified first by CC (chloroform/ethyl acetate 70:30); yield: quant. (0.61 g); ^1^H NMR (CD_3_OD): 3.72 (s, 3H), 3.79 (s, 3H), 5.95 (s, 1H), 6.83 (dd, *J* = 8.8 Hz, *J* = 2.5 Hz, 1H) 7.02-7.05 (m, 3H), 7.25 (t, *J* = 8.2 Hz, 1H), 7. 6 (d, *J* = 2.5 Hz, 1H), 7.76 (d, *J* = 8.8 Hz, 1H); ^13^C NMR (CD_3_DO): 55.0, 55.6, 72.5, 104.8, 112.1, 112.9, 115.2, 118.7, 123.0, 129.4, 135.8, 143.9, 147.2, 147.3, 156.9, 159.2;

**(6-Methoxy-1,3-benzothiazol-2-yl)(4-methoxyphenyl)methanol** (**9ii**). The title compound was prepared by reaction of 6-methoxy-1,3-benzothiazole (**6iii**) (0.5 g, 3.03 mmol), *n-*BuLi (2,5 M in hexane; 1.2 mL, 3.03 mmol) and 4-methoxybenzaldehyde (0.3 mL, 2.32 mmol) according to method A. The product was purified by CC (chloroform/ethyl acetate 70:30); yield: quant. (0.61 g); ^1^H NMR (CDCl_3_): 3.79 (s, 3H), 3.85 (s, 3H), 6.06 (s, 1H), 6.88-6.90 (m, 2H), 7.04 (dd, *J* = 9.1 Hz, *J* = 2.5 Hz, 1H), 7.26 (d, *J* = 2.5 Hz, 1H), 7.42 (d, *J* = 8.8 Hz, 2H), 7.84 (d, *J* = 9.1 Hz, 1H); ^13^C NMR (CDCl_3_): 55.3, 55.8, 73.9, 104.3, 114.2, 115.5, 116.5, 117.0, 123.4, 128.1, 133.2, 136.6, 146.9, 157.6, 159.8, 172.6;

**1,3-Benzothiazol-2-yl(3-methoxyphenyl)methanol** (**12ii**)**.** The title compound was prepared by reaction of 1,3-benzothiazole (0.20 mL, 1.85 mmol), *n-*BuLi (2,5 M in hexane; 0.74 mL, 1.85 mmol) and 3-methoxybenzaldehyde (0.2 mL, 1.42 mmol) according to method A. The product was purified by CC (hexane/ethyl acetate 80:20); yield: 91 % (0.35 g); ^1^H NMR CDCl_3_: 3.92 (s, 3H), 6.26 (s, 1H), 6.97-7.03 (m, 1H), 7.24-7.26 (m, 2H), 7.42 (t, *J* = 8.2 Hz, 1H), 7.48-7.51 (m, 1H), 7.57-7.60 (m, 1H), 7.96 (d, *J* = 8.5 Hz, 1H), 8.10 (d, *J* = 8.2 Hz, 1H); ^13^C NMR (CDCl_3_): 55.2, 74.1, 112.0, 114.3, 119.0, 121.7, 123.0, 125.1, 126.1, 129.8, 135.2, 142.4, 152.4, 159.9, 175.1;

**6-Methoxy-1,3-benzothiazole-2-carboxylic acid (2-methoxyphenyl)amide** (**15i**). The title compound was prepared by reaction of 6-methoxy-1,3-benzothiazole (**6iii**) (0.20 g, 1.21 mmol), *n-*BuLi (2,5 M in hexane; 0.5 mL, 1.21 mmol) and 1-isocyanato-2-methoxybenzene (0.1 mL, 0.93 mmol) according to method A. The product was purified by CC (hexane/ethyl acetate 80:20); yield: quant. (0.25 g); ^1^H NMR (CD_3_OD): 3.92 (s, 3H), 4.0 (s, 3H), 6.95 (dd, *J* = 8.2 Hz, *J* = 1.3 Hz, 1H), 7.03 (dd, *J* = 8.2 Hz, *J* = 1.2 Hz, 1H), 7.10 (dq, *J* = 8.2 Hz, *J* = 1.6 Hz, *J* = 0.6 Hz, 1H), 7.17 (dd, *J* = 8.8 Hz, *J* = 2.5 Hz, 1H), 7.40 (d, *J* = 2.5 Hz, 1H), 8.03 (d, *J* = 9.1 Hz, 1H), 8.51 (dd, *J* = 7.9 Hz, *J* = 1.6 Hz, 1H), 9.76 (s, 1H); ^13^C NMR (CD_3_OD): 55.9, 56.0, 104.3, 110.5, 117.1, 120.1, 120.3, 121.3, 124.5, 125.2, 127.3, 139.3, 147.7, 148.9, 159.3, 162.0;

**6-Methoxy-1,3-benzothiazole-2-carboxylic acid (3-methoxyphenyl)amide** (**17i**). The title compound was prepared by reaction of 6-methoxy-1,3-benzothiazole (**6iii**) (0.25 g, 1.51 mmol), *n-*BuLi (2,5 M in hexane; 0.6 mL, 1.51 mmol) and 1-isocyanato-3-methoxybenzene (0.2 mL, 1.16 mmol) according to method A. The product was purified by CC (hexane/ethyl acetate 80:20); yield: quant. (0.25 g); the compound was used for the next step without characterization.

**(6-Methoxy-1,3-benzothiazol-2-yl)(3-methoxyphenyl)methanone** (**6i**)**.** The title compound was prepared by reaction of (6-methoxy-1,3-benzothiazol-2-yl)(3-methoxyphenyl)methanol (**6ii**) (0.35 g, 1.16 mmol) and SIBX (0.65 g, 2.32 mmol) according to method C. The product was purified by CC (hexane/ethyl acetate 95:5); yield: quant. (0.35 g); ^1^H NMR (CD_3_OD): 3.91 (s, 3H), 3.93 (s, 3H), 7.17-7.22 (m, 2H), 7.41 (d, *J* = 2.5 Hz, 1H), 7.46 (t, *J* = 7.8 Hz, 1H), 8.03-8.04 (m, 1H), 8.10 (d, *J* = 9.1 Hz, 1H), 8.19-8.22 (m, 1H); ^13^C NMR (CD_3_DO): 55.5, 55.9, 103.4, 115.2, 117.6, 120.3, 124.1, 126.5, 129.5, 136.4, 139.1, 148.5, 159.6, 159.8, 164.6, 184.9; MS (ESI): 299.9 (M+H)^+^;

**2-[(4-Hydroxyphenyl)(methylsulfanyl)methyl]-1,3-benzothiazol-6-ol** (**9**). The title compound was prepared by reaction of (6-methoxy-1,3-benzothiazol-2-yl)(4-methoxyphenyl)methanol (**9ii**) (0.30 g, 1.00 mmol) and boron trifluoride methyl sulfide complex in dichloromethane (1 M, 15.8 mL, 150.00 mmol) according to method D. The product was purified by CC (hexane/ethyl acetate 50:50); yield: 86 % (0.26 g); ^1^H NMR (CD_3_OD): 2.10 (s, 3H), 5.36 (s, 1H), 6.77-6.79 (m, 2H), 6.97 (dd, *J* = 8.8 Hz, *J* = 2.5 Hz, 1H), 7.27 (d, *J* = 1.9 Hz, 1H), 7.31-7.33 (m, 2H), 7.72 (d, *J* = 8.5 Hz, 1H); ^13^C NMR (CD_3_OD): 14.4, 56.7, 107.5, 114.9, 115.5, 116.8, 123.0, 131.1, 131.3, 131.5, 136.6, 145.4, 155.1, 156.9, 168.7; MS (ESI): 302.6 (M+H)^+^;

**6-Methoxy-2-(4-hydroxybenzyl)-1,3-benzothiazole** (**10**)**.** A solution of NaI (0.89 g, 5.97 mmol) and trimethylsilyl chloride (0.8 mL, 5.97 mmol) in acetonitrile was stirred at room temperature for 15 min. The reaction mixture was cooled to 0 °C, and a solution of (6-methoxy-1,3-benzothiazol-2-yl)(4-methoxyphenyl)methanol (**9ii**) (0.20 g, 0.66 mmol) in acetonitrile was added drop-wise during 15 min. The reaction mixture was then refluxed overnight. To quench the reaction a solution of NaHCO_3_ was slowly added at room temperature, followed by a solution of Na_2_S_2_O_3_**·**5H_2_O. The aqueous solution was then extracted three times with ethyl acetate. The combined organic layers were washed with brine, dried over magnesium sulfate, filtered and concentrated to dryness. The product was purified by CC (hexane/ethyl acetate 80:20); yield: 28 % (0.05 g); ^1^H NMR (CDCl_3_): 3.84 (s, 3H), 4.31 (s, 2H), 6.76-6.79 (m, 2H), 7.04 (dd, *J* = 8.8 Hz, *J* = 2.5 Hz, 1H), 7.16-7.19 (m, 2H), 7.24 (d, *J* = 2.5 Hz, 1H), 7.86 (d, *J* = 9.1 Hz, 1H); ^13^C NMR (CDCl_3_): 39.3, 55.8, 104.3, 114.3, 115.3, 115.9, 122.8, 128.7, 130.2, 130.3, 136.5, 147.0, 155.5, 157.5, 170.2; MS (ESI): 272.5 (M+H)^+^;

**6-Methoxy-2-(4-methoxybenzyl)-1,3-benzothiazole** (**11i**)**.** A solution of NaI (0.89 g, 5.97 mmol) and trimethylsilyl chloride (0.8 mL, 5.97 mmol) in acetonitrile was stirred at room temperature for 15 min. The reaction mixture was cooled at 0 °C, and a solution of (6-methoxy-1,3-benzothiazol-2-yl)(4-methoxyphenyl)methanol (**9ii**) (0.20 g, 0.66 mmol) in acetonitrile was added drop-wise during 15 min. The reaction mixture was then refluxed overnight. To quench the reaction a solution of NaHCO_3_ was slowly added at room temperature, followed by a solution of Na_2_S_2_O_3_**·**5H_2_O. The aqueous solution was then extracted three times with ethyl acetate. The combined organic layers were washed with brine, dried over magnesium sulfate, filtered and concentrated to dryness. The product was purified by CC (hexane/ethyl acetate 80:20); yield: 56 % (0.11 g); ^1^H NMR (CDCl_3_): 3.79 (s, 3H), 3.83 (s, 3H), 4.33 (s, 2H), 6.86-6.89 (m, 2H), 7.04 (dd, *J* = 8.8 Hz, *J* = 2.5 Hz, 1H), 7.23 (d, *J* = 2.5 Hz, 1H), 7.26-7.29 (m, 2H), 7.86 (d, *J* = 9.1 Hz, 1H); ^13^C NMR (CDCl_3_): 39.6, 55.2, 55.7, 104.2, 114.2, 115.0, 123.1, 125.7, 129.4, 130.2, 136.8, 147.6, 157.4, 158.8, 163.8, 169.3;

**1,3-Benzothiazol-2-yl(3-methoxyphenyl)methanone** (**12i**). The title compound was prepared by reaction of 1,3-benzothiazol-2-yl(3-methoxyphenyl)methanol (**12ii**) (0.35 g, 1.29 mmol) and SIBX (0.72 g, 2.58 mmol) according to method C. The product was purified by CC (hexane/ethyl acetate 90:10); yield: 61 % (0.215 g); ^1^H NMR CDCl_3_: 3.92 (s, 3H), 7.23 (dd, *J* = 8.2 Hz, *J* = 2.8 Hz, 1H), 7.48 (t, *J* = 8.2 Hz, 1H), 7.54-7.61 (m, 2H), 8.02-8.06 (m, 2H), 8.24 (t, *J* = 8.2 Hz, 2H); ^13^C NMR (CDCl_3_): 55.5, 115.3, 120.6, 122.2, 124.2, 125.8, 126.9, 127.6, 129.5, 136.2, 137.0, 153.9, 159.6, 167.1, 185.1;

**2-[(3-Hydroxyphenyl)hydroxymethyl]-1,3-benzothiazol-6-ol**. (**7**). The title compound was prepared by reaction of (6-methoxy-1,3-benzothiazol-2-yl)(3-methoxyphenyl)methanone (**6i**) (0.17 g, 0.57 mmol) and boron trifluoride methyl sulfide complex in dichloromethane (1 M, 9.0 mL, 85.55 mmol) according to method D. The product was purified by preparative HPLC; yield: 16 % (0.02 g). ^1^H NMR (CD_3_OD): 5.84 (s, 1H), 6.61 (dd, *J* = 2.5 Hz, *J* = 0.9 Hz, 1H), 6.84-6.87 (m, 3H), 7.06 (t, *J* = 7.9 Hz, 1H), 7.17 (d, *J* = 2.5 Hz, 1H), 7.59 (d, *J* = 8.8 Hz, 1H); ^13^C NMR (CD_3_OD): 66.8, 75.0, 107.6, 114.6, 116.1, 116.8, 119.0, 123.8, 130.6, 144.6, 147.6, 156.9, 158.7, 175.6; MS (ESI): 274.0 (M+H)^+^;

**2-[(3-Hydroxyphenyl)(methoxy)methyl]-1,3-benzothiazol-6-ol**. (**8**). The title compound was prepared by reaction of (6-methoxy-1,3-benzothiazol-2-yl)(3-methoxyphenyl)methanone (**6i**) (0.17 g, 0.57 mmol) and boron trifluoride methyl sulfide complex in dichloromethane (1 M, 9.0 mL, 85.55 mmol) according to method D. The product was purified by preparative HPLC; yield: 16 % (0.02 g). ^1^H NMR (CD_3_OD): 3.35 (s, 3H), 5.42 (s, 1H), 6.63-6.65 (m, 1H), 6.80-6.87 (m, 3H), 7.08 (t, *J* = 7.8 Hz, 1H), 7.17 (d, *J* = 2.2 Hz, 1H), 7.61 (d, *J* = 8.8 Hz, 1H); ^13^C NMR (CD_3_OD): 57.8, 84.3, 107.6, 114.9, 116.5, 116.9, 119.4, 124.0, 130.8, 137.6, 141.9, 147.5, 157.1, 158.9, 172.5; MS (ESI): 288.0 (M+H)^+^;

**6-Hydroxy-2-(4-hydroxybenzyl)-1,3-benzothiazole** (**11**)**.** The title compound was prepared by reaction of 6-methoxy-2-(4-methoxybenzyl)-1,3-benzothiazole (**11i**) (0.11 g, 0.37 mmol) and pyridinium hydrochloride (4.29 g, 37.10 mmol) according to method E. The product was purified by recrystallisation (hexane/ethyl acetate); yield: 47 % (0.05 g); ^1^H NMR (CD_3_OD): 4.27 (s, 2H), 6.76-6.80 (m, 2H), 6.96 (dd, *J* = 8.8 Hz, *J* = 2.5 Hz, 1H), 7.16-7.18 (m, 2H), 7.21 (d, *J* = 2.5 Hz, 1H), 7.71 (d, *J* = 8.8 Hz, 1H); ^13^C NMR (CD_3_OD): 39.9, 107.6, 116.6, 116.7, 118.5, 118.8, 123.4, 129.5, 131.2, 137.6, 137.9, 157.3, 163.7, 171.6; MS (ESI): 258.5 (M+H)^+^;

**6-Methoxy-1,3-benzothiazole-2-carboxylic acid (2-hydroxyphenyl)amide** (**15**). The title compound was prepared by reaction of 6-methoxy-1,3-benzothiazole-2-carboxylic acid (2-methoxyphenyl)amide (**15i**) (0.10 g, 0.32 mmol) and boron trifluoride methyl sulfide complex in dichloromethane (1 M, 5.0 mL, 47.72 mmol) according to method D. The product was purified by recrystallisation (ethyl acetate); yield: 21 % (0.02 g). ^1^H NMR (CD_3_COCD_3_): 3.94 (s, 3H), 6.90-6.95 (m, 1H), 7.02-7.03 (m, 2H), 7.24 (dd, *J* = 9.1 Hz, *J* = 2.7 Hz, 1H), 7.73 (d, *J* = 2.4 Hz, 1H), 8.05 (d, *J* = 9.1 Hz, 1H), 8.36 (d, *J* = 8.2, 1H), 9.38 (s, 1H), 9.87 (s, 1H); ^13^C NMR (CD_3_COCD_3_): 56.8, 105.7, 116.5, 118.9, 121.4, 121.4, 126.2, 126.4, 127.5, 140.4, 148.0, 148.7, 158.8, 160.8, 162.9; MS (ESI): 301.1 (M+H)^+^;

**6-Hydroxy-1,3-benzothiazole-2-carboxylic acid (2-hydroxyphenyl)amide** (**16**). The title compound was prepared by reaction of 6-methoxy-1,3-benzothiazole-2-carboxylic acid (2-methoxyphenyl)amide (**15i**) (0.1 g, 0.32 mmol) and boron trifluoride methyl sulfide complex in dichloromethane (1 M, 5.0 mL, 47.72 mmol) according to method D. The product was purified by recrystallisation (ethyl acetate); yield: 11 % (0.01 g). ^1^H NMR (CD_3_COCD_3_): 6.90-6.95 (m, 1H), 7.02-7.03 (m, 2H), 7.20 (dd, *J* = 8.8 Hz, *J* = 2.2 Hz, 1H), 7.54 (d, *J* = 2.5 Hz, 1H), 7.99 (d, *J* = 8.8 Hz, 1H), 8.30 (m, 1H); ^13^C NMR (CD_3_COCD_3_): 107.7, 115.9, 118.3, 120.8, 120.9, 125.6, 126.1, 126.9, 139.9, 147.3, 147.7, 158.1, 158.3, 161.6; MS (ESI): 287.0 (M+H)^+^;

**6-Methoxy-1,3-benzothiazole-2-carboxylic acid (3-hydroxyphenyl)amide** (**17**). The title compound was prepared by reaction of 6-methoxy-1,3-benzothiazole-2-carboxylic acid (3-methoxyphenyl)amide (**17i**) (0.10 g, 0.32 mmol) and boron trifluoride methyl sulfide complex in dichloromethane (1 M, 5.0 mL, 47.72 mmol) according to method D. The product was purified by recrystallisation (ethyl acetate); yield: 21 % (0.02 g). ^1^H NMR (CD_3_COCD_3_): 3.94 (s, 3H), 6.67 (dd, *J* = 2.1 Hz, *J* = 0.9, Hz, 1H), 7.2 (m, 2H), 7.36 (m, 1H), 7.36 (t, *J* = 2.1 Hz, 1H), 7.59-7.61 (m, 1H), 7.99 (d, *J* = 8.8 Hz, 1H), 8.83 (s, 1H), 9.86 (s, 1H); ^13^C NMR (CD_3_COCD_3_): 57.3, 106.2, 109.2, 113.2, 113.5, 119.3, 126.8, 131.5, 140.8, 141.0, 149.3, 159.8, 159.8, 161.2, 163.6; MS (ESI): 301.0 (M+H)^+^;

**6-Hydroxy-1,3-benzothiazole-2-carboxylic acid (3-hydroxyphenyl)amide** (**18**). The title compound was prepared by reaction of 6-methoxy-1,3-benzothiazole-2-carboxylic acid (3-methoxyphenyl)amide (**17i**) (0.10 g, 0.32 mmol) and boron trifluoride methyl sulfide complex in dichloromethane (1 M, 5.0 mL, 47.72 mmol) according to method D. The product was purified by recrystallisation (ethyl acetate); yield: 11 % (0.01 g). ^1^H NMR (CD_3_COCD_3_): 6.67 (dd, *J* = 2.1 Hz, *J* = 0.9, Hz, 1H), 7.19-7.21 (m, 2H), 7.35-7.37 (m, 1H), 7.55 (d, *J* = 2.1 Hz, 1H), 7.59 (t, *J* = 2.1 Hz, 1H), 7.95 (d, *J* = 8.8 Hz, 1H), 8.83 (s, 2H), 9.83 (s, 1H); ^13^C NMR (CD_3_COCD_3_): 108.7, 109.1, 113.2, 113.4, 119.2, 127.0, 131.5, 140.9, 141.1, 148.7, 159.1, 159.8, 159.9, 162.9; MS (ESI): 287.0 (M+H)^+^;

**3-Methoxy-*N*-(6-methoxy-1,3-benzothiazol-2-yl)benzenesulfonamide** (**22i**). The title compound was prepared by reaction of 6-methoxy-1,3-benzothiazol-2-ylamine (0.3 g, 1.66 mmol) and 3-methoxybenzenesulfonyl chloride (0.3 mL, 1.66 mmol) according to method B. The product was purified by recrystallisation (methanol); yield: 89 % (0.66 g); ^1^H NMR (CD_3_COCD_3_): 3.84 (s, 3H), 3.85 (s, 3H), 7.00 (dd, *J* = 2.5 Hz, *J* = 8.8 Hz, 1H), 7.13 (ddd, *J* = 7.9 Hz, *J* = 2.5 Hz, *J* = 0.9 Hz, 1H), 7.34 (d, *J* = 8.8 Hz, 1H), 7.38 (d, *J* = 2.5 Hz, 1H), 7.41-7.43 (m, 1H), 7.45 (d, *J* = 8.2 Hz, 1H), 7.48 (dt, *J* = 7.9 Hz, *J* = 1.3 Hz, 1H); ^13^C NMR (CD_3_COCD_3_): 56,0; 56,2; 107,5; 112,3; 114,4; 115,6; 118,6; 119,1; 130,7; 130,8; 133,2; 134,3; 136,1; 145,1; 157,8;

**1-(6-Methoxy-1,3-benzothiazol-2-yl)-3-(3-methoxyphenyl)urea** (**23i**). The title compound was prepared by reaction of 6-methoxy-1,3-benzothiazol-2-ylamine (0.33 g, 1.85 mmol) and 1-isocyanato-3-methoxybenzene (0.2 mL, 1.85 mmol) according to method B. The product was purified by recrystallisation (methanol); yield: quant. (0.61 g); ^1^H NMR (CD_3_COCD_3_): 3.78 (s, 3H), 3.82 (s, 3H), 6.64 (dd, *J* = 8.2 Hz, *J* = 2.2 Hz, 1H), 6.99 (dd, *J* = 8.8 Hz, *J* = 2.5 Hz, 1H), 7.03-7.05 (m, 1H), 7.21-7.25 (m, 2H), 7.50 (d, *J* = 2.5 Hz, 1H), 7.56 (d, *J* = 8.8 Hz, 1H); ^13^C NMR (CD_3_COCD_3_): 56,2; 56,8; 105,9; 106,1; 109,5; 112,3; 115,6; 121,5; 121,5; 131,0; 134,0; 134,0; 141,2; 157,2; 161,3; 206,2;

**1-(6-Methoxy-1,3-benzothiazol-2-yl)-3-(3-methoxyphenyl)thiourea** (**24i**). The title compound was prepared by reaction of 6-methoxy-1,3-benzothiazol-2-ylamine (0.30 g, 1.66 mmol) and 1-isothiocyanato-3-methoxybenzene (0.23 mL, 1.66 mmol) according to method B. The product was purified by CC (chloroform/methanol 95:05); yield: 30 % (0.17 g); MS (ESI): 346 (M+H)^+^;

***N*-(6-Methoxy-1,3-benzothiazol-2-yl)-2-(3-methoxyphenyl)acetamide** (**25i**). The title compound was prepared by reaction of 6-methoxy-1,3-benzothiazol-2-ylamine (0.3 g, 1.66 mmol) and (3-methoxyphenyl)acetyl chloride (0.26 mL, 1.66 mmol) according to method B. The product was purified by CC (chloroform/methanol 95:05); yield: 82 % (0.45 g); MS (ESI): 329 (M+H)^+^;

**4-Methoxy-*N*-(6-hydroxy-1,3-benzothiazol-2-yl)benzamide** (**19**). The title compound was prepared by reaction of 4-methoxy-*N*-(6-methoxy-1,3-benzothiazol-2-yl)benzamide (**19i**) (0.5 g, 1.59 mmol) and boron trifluoride methyl sulfide complex in dichloromethane (1 M, 25.1 mL, 239.00 mmol) according to method D. The product was purified by preparative HPLC; yield: 23 % (0.11 g). ^1^H NMR (CD_3_OD): 3.94 (s, 3H), 7.12-7.16 (m, 3H), 7.40 (d, *J* = 2.5 Hz, 1H), 7.78 (d, *J* = 9.1 Hz, 1H), 8.30-8.32 (m, 2H); ^13^C NMR (CD_3_OD): 56.4, 109.2, 115.5, 118.4, 118.5, 118.6, 119.8, 124.4, 130.2, 134.2, 133.4, 158.0, 166.4, 168.2, 173.3; MS (ESI): 300.1 (M+H)^+^;

**4-Hydroxy-*N*-(6-hydroxy-1,3-benzothiazol-2-yl)benzamide** (**20**). The title compound was prepared by reaction of 4-methoxy-*N*-(6-methoxy-1,3-benzothiazol-2-yl)benzamide (**19i**) (0.5 g, 1.59 mmol) and boron trifluoride methyl sulfide complex in dichloromethane (1 M, 25.1 mL, 239.00 mmol) according to method D. The product was purified by preparative HPLC; yield: 13 % (0.06 g). ^1^H NMR (CD_3_OD): 6.80-6.83 (m, 2H), 6.98 (dd, *J* = 8.8 Hz, *J* = 2.5 Hz, 1H), 7.22 (d, *J* = 2.2 Hz, 1H), 7.64 (d, *J* = 9.1 Hz, 1H), 8.10-8.12 (m, 2H); ^13^C NMR (CD_3_OD): 107.7, 115.9, 118.3, 120.8, 120.9, 125.6, 126.1, 126.9, 139.9, 147.3, 147.7, 158.1, 158.3, 161.6; MS (ESI): 287.6 (M+H)^+^;

**3-Hydroxy-*N*-(6-hydroxy-1,3-benzothiazol-2-yl)benzamide** (**21**). The title compound was prepared by reaction of 3-methoxy-*N*-(6-methoxy-1,3-benzothiazol-2-yl)benzamide (**21i**) (0.5 g, 1.59 mmol) and boron trifluoride methyl sulfide complex in dichloromethane (1 M, 25.0 mL, 239.00 mmol) according to method D. The product was purified by recrystallisation (ethyl acetate); yield: quant. (0.46 g). ^1^H NMR (CD_3_OD): 6.83 (dd, *J* = 8.5 Hz, *J* = 2.5 Hz, 1H), 6.95 (dd, *J* = 2.5 Hz, *J* = 0.9 Hz, 1H), 7.14 (d, *J* = 2.2 Hz, 1H), 7.26 (t, *J* = 8.2 Hz, 1H), 7.32 (t, *J* = 2.2 Hz, 1H), 7.37-7.39 (m, 1H), 7.48 (d, *J* = 8.8 Hz, 1H); ^13^C NMR (CD_3_OD): 107.2, 112.3, 115.9, 116.5, 119.9, 121.0, 122.4, 127.5, 131.0, 134.7, 135.2, 146.6, 155.9, 159.2; MS (ESI): 286.9 (M+H)^+^;

**3-Hydroxy-*N*-(6-hydroxy-1,3-benzothiazol-2-yl)-benzenesulfonamide** (**22**). The title compound was prepared by reaction of 3-methoxy-*N*-(6-methoxy-1,3-benzothiazol-2-yl)-benzenesulfonamide (**22i**) (0.52 g, 1.48 mmol) and boron tribromide in dichloromethane (1 M, 14.8 mL, 14.80 mmol) according to method F. The product was purified by recrystallisation (H_2_O:methanol); yield: 88 % (0.42 g); ^1^H NMR (CD_3_SOCD_3_): 6.82 (dd, *J* = 2.5 Hz, *J* = 8.8 Hz, 1H), 6.96 (dd, *J* = 8.2 Hz, *J* = 2.2 Hz, 1H), 7.12 (d, *J* = 8.5 Hz, 1H), 7.18 (d, *J* = 2.5 Hz, 1H), 7.21-7.26 (m, 2H), 7.33 (t, *J* = 8.0 Hz, 1H), 9.63 (s, 1H), 10.00 (s, 1H), 12.9 (s, 1H); ^13^C NMR (CD_3_SOCD_3_): 108,4; 112,2; 113,4; 115,2; 116,2; 119,1; 124,8; 125,8; 130,0; 139,1; 143,2; 154,1; 157,5; MS (ESI): 323.1 (M+H)^+^;

**1-(6-Hydroxy-1,3-benzothiazol-2-yl)-3-(3-hydroxyphenyl)urea** (**23**). The title compound was prepared by reaction of 1-(6-methoxy-1,3-benzothiazol-2-yl)-3-(3-methoxyphenyl)urea (**23i**) (0.60 g, 1.82 mmol) and boron tribromide in dichloromethane (1 M, 18.2 mL, 18.20 mmol) according to method F. The product was purified by CC (chloroform/methanol 90:10); yield: 64 % (0.35 g); ^1^H NMR (CD_3_SOCD_3_): 6.46 (dd, *J* = 1.5 Hz, *J* = 8.2 Hz, 1H), 6.84-6.87 (m, 2H), 7.08 (t, *J* = 7.9 Hz, 1H), 7.14 (t, *J* = 2.1 Hz, 1H), 7.24 (d, *J* = 2.4 Hz, 1H), 7.46 (d, *J* = 8.8 Hz, 1H), 9.01 (s, 1H), 9.39 (s, 1H), 9.41 (s, 1H), 10.48 (s, 1H); ^13^C NMR (CD_3_SOCD_3_): 105,8; 106,5; 109,3; 110,0; 114,8; 120,1; 120,2; 129,6; 132,6; 139,7; 140,9; 153,9; 158,0; 206,5; MS (ESI): 302.1 (M+H)^+^;

**1-(6-Hydroxy-1,3-benzothiazol-2-yl)-3-(3-hydroxyphenyl)thiourea** (**24**). The title compound was prepared by reaction of 1-(6-methoxy-1,3-benzothiazol-2-yl)-3-(3-methoxyphenyl)thiourea (**24i**). (0.17 g, 0.49 mmol) and boron tribromide in dichloromethane (1 M, 4.9 mL, 4.90 mmol) according to method F. The product was purified by CC (chloroform/methanol 90:10); yield: 26 % (0.04 mg); ^1^H NMR (CD_3_SOCD_3_): 6.53-6.56 (m, 1H), 6.87 (dd, *J* = 8.5 Hz, *J* = 2.3, 1H), 7.09-7.22 (m, 4H), 7.39-7.40 (m, 1H), 8.30 (s, 1H), 9.41 (s, 1H), 9.48 (s, 1H), 9.58 (s, 1H); ^13^C NMR (CD_3_SOCD_3_): 115,2; 115,8; 116,1; 116,23; 116,7; 116,8; 116,9; 117,0; 117,4; 117,6; 117,7; 125,2; 125,6; 157,3; MS (ESI): 318.1 (M+H)^+^;

***N*-(6-Hydroxy-1,3-benzothiazol-2-yl)-2-(3-hydroxyphenyl)acetamide** (**25**). The title compound was prepared by reaction of *N*-(6-methoxy-1,3-benzothiazol-2-yl)-2-(3-methoxy-phenyl)acetamide (**25i**). (0.45 g, 1.37 mmol) and boron tribromide in dichloromethane (1 M, 13.7 mL, 13.70 mmol) according to method F. The product was purified by preparative HPLC; yield: 36 % (0.15 g); ^1^H NMR (CD_3_SOCD_3_): 3.69 (s, 2H), 6.66 (dd, *J* = 8.2 Hz, *J* = 2.2, *J* = 0.9, 1H), 6.74-6.77 (m, 2H), 6.88 (dd, *J* = 8.8 Hz, *J* = 2.5, 1H), 7.11 (t, *J* = 7.7, 1H), 7.26 (d, *J* = 2.2, 1H), 7.54 (d, *J* = 8.8 Hz, 1H), 12.3 (s, 1H); ^13^C NMR (CD_3_SOCD_3_): 41,8; 106,4; 113,8; 115,2; 116,0; 119,8; 121,0; 129,3; 132,7; 136,0; 141,5; 154,1; 154,9; 157,3; 169,7; MS (ESI): 301.1 (M+H)^+^;

**Biological Methods.** [2, 4, 6, 7-^3^H]-E2 and [2, 4, 6, 7-^3^H]-E1 were bought from Perkin Elmer, Boston. Quickszint Flow 302 scintillator fluid was bought from Zinsser Analytic, Frankfurt.

17β-HSD1 and 17β-HSD2 were obtained from human placenta according to previously described procedures. Fresh human placenta was homogenized and cytosolic fraction and microsomes were separated by centrifugation. For the partial purification of 17β-HSD1, the cytosolic fraction was precipitated with ammonium sulfate. 17β-HSD2 was obtained from the microsomal fraction.

**Inhibition of 17β-HSD1.** Inhibitory activities were evaluated by an established method with minor modifications. Briefly, the enzyme preparation was incubated with NADH [500 µM] in the presence of potential inhibitors at 37 °C in a phosphate buffer (50 mM) supplemented with 20 % of glycerol and EDTA (1mM)**.** Inhibitor stock solutions were prepared in DMSO. The final concentration of DMSO was adjusted to 1 % in all samples. The enzymatic reaction was started by addition of a mixture of unlabelled- and [2, 4, 6, 7-^3^H]-E1 (final concentration: 500 nM, 0.15 µCi). After 10 min, the incubation was stopped with HgCl_2_ and the mixture was extracted with diethylether. After evaporation, the steroids were dissolved in acetonitrile. E1 and E2 were separated using acetonitrile/water (45:55) as mobile phase in a C18 reverse phase chromatography column (Nucleodur C18 Gravity, 3 µm, Macherey-Nagel, Düren) connected to a HPLC-system (Agilent 1100 Series, Agilent Technologies, Waldbronn). Detection and quantification of the steroids were performed using a radioflow detector (Berthold Technologies, Bad Wildbad). The conversion rate was calculated after analysis of the resulting chromatograms according to the following equation: % conversion = % E2/(% E2 + % E1) × 100. Each value was calculated from at least three independent experiments.

**Inhibition of 17β-HSD2.** The 17β-HSD2 inhibition assay was performed similarly to the 17β-HSD1 procedure. The microsomal fraction was incubated with NAD^+^ [1500 µM], test compound and a mixture of unlabelled- and [2, 4, 6, 7-^3^H]-E2 (final concentration: 500 nM, 0.11 µCi) for 20 min at 37 °C. Further treatment of the samples and HPLC separation were carried out as mentioned above.

The conversion rate was calculated after analysis of the resulting chromatograms according to the following equation: % conversion = % E1/(% E1 + % E2) × 100.

**ER affinity.** The binding affinity of selected compounds to the ERα and ERβ was determined according to Zimmermann et al. Briefly, 0.25 pmol of ERα or ERβ, respectively, were incubated with [2, 4, 6, 7-^3^H]-E2 (10 nM) and test compound for 1 h at room temperature. The potential inhibitors were dissolved in DMSO (5 % final concentration). Evaluation of non-specific-binding was performed with diethylstilbestrol (10 µM). After incubation, ligand-receptor complexes were selectively bound to hydroxyapatite (5 g/ 60 mL TE-buffer). The complex formed was separated, washed and resuspended in ethanol. For radiodetection, scintillator cocktail (Quickszint 212, Zinsser Analytic, Frankfurt) was added and samples were measured in a liquid scintillation counter (Rack Beta Primo 1209, Wallac, Turku). For determination of the relative binding affinity (RBA), inhibitor and E2 concentrations required to displace 50 % of the receptor bound labelled E2 were determined. RBA values were calculated according to the following equation: RBA [%] = IC_50_ (E2) / IC_50_ (compound) × 100. The RBA value for E2 was arbitrarily set at 100 %.

**Inhibition of 17β-HSD1 in T47-D cells.** T47-D cells were obtained from ECACC, Salisbury. Stripped FCS and cell culture media were purchased from CCpro, Oberdorla. A stock culture of T47-D cells was grown in RPMI 1640 medium supplemented with 10 % FCS, L-glutamine (2 mM), penicillin (100 IU/mL), streptomycin (100 µg/mL), insulin-zinc-salt (10 µg/mL) and sodium pyruvate (1 mM) at 37 °C under 5 % CO_2_ humidified atmosphere.

The cells were seeded into a 24-well plate at 1x10^6^ cells/well in DMEM medium with FCS, L-glutamine and the antibiotics added in the same concentrations as mentioned above. After 24 h the medium was changed for fresh serum free DMEM, and a solution of test compound in DMSO was added. Final concentration of DMSO was adjusted to 1 % in all samples. After a pre-incubation of 30 min at 37°C with 5 % CO_2_, the incubation was started by addition of a mixture of unlabelled- and [2, 4, 6, 7-^3^H]- E1 (final concentration : 50 nM, 0.15 µCi). After 0.5 h incubation, the enzymatic reaction was stopped by removing of the supernatant medium. The steroids were extracted into diethylether. Further treatment of the samples was carried out as mentioned for the 17β-HSD1 assay.

**References**

Kishore Kumar GD, Natarajan A (2008) Total synthesis of ovalifoliolatin B, acerogenins A and C. Tetrahedron Letters 49: 2103-2105.

Obase H, Tatsuno H, Goto K, Shigenobu K, Kasuya Y et al. (1978) Synthesis and adrenergic β-blocking activity of some propanolamine derivatives. Chem Pharm Bull 26: 1443-1452.

Muramoto H, Fukuda K, Hasegawa T, Okamoto K, Kotani T (1996) Preparation of hypolipemic aroylbenzothiazoles. Eur Pat Appl EP0735029 (A1)

Myllymaeki MJ, Saario SM, Kataja AO, Castillo-Melendez JA, Nevalainen T et al. (2007) Design, synthesis, and in vitro evaluation of carbamate derivatives of 2-benzoxazolyl- and 2-benzothiazolyl-(3-hydroxyphenyl)-methanones as novel fatty acid amide hydrolase inhibitors. J Med Chem 50: 4236-4242.

Parlati F, Ramesh UV, Singh R, Payan DG, Lowe R et al. (2005) Benzothiazoles and thiazolo`5,5-b!pyridines as ubiquitin ligase inhibitors, their preparation and pharmaceutical compositions. PCT Int Appl WO2005037845 (A1).
